# Synthesis and Characterization of Superparamagnetic Iron Oxide Nanoparticles: A Series of Laboratory Experiments

**DOI:** 10.1021/acs.jchemed.3c00996

**Published:** 2024-04-09

**Authors:** Armando D. Urbina, Hari Sridhara, Alexis Scholtz, Andrea M. Armani

**Affiliations:** † Mork Family Department of Chemical Engineering and Materials Science, University of Southern California, Los Angeles, California 90089, United States; ‡ Alfred E. Mann Department of Biomedical Engineering, University of Southern California, Los Angeles, California 90089, United States; § Ellison Institute of Technology, Los Angeles, California 90064, United States

**Keywords:** Upper-Division Undergraduate, Inorganic Chemistry, Laboratory Instruction, Hands-On Learning, Inquiry-Based Learning, Magnetic Properties, Physical Properties, Materials Science, Metals, Nanotechnology

## Abstract

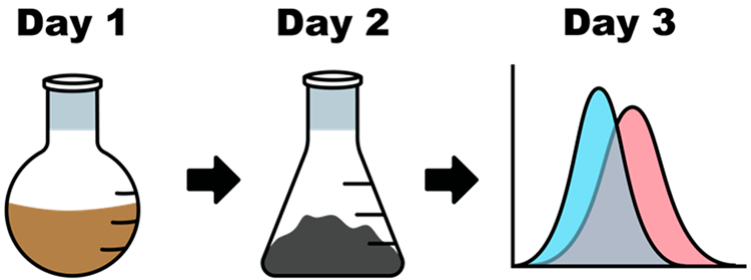

The following laboratory procedure provides students with hands-on experience in nanomaterial chemistry and characterization. This three-day protocol is easy to follow for undergraduates with basic chemistry or materials science backgrounds and is suitable for inclusion in upper-division courses in inorganic chemistry or materials science. Students use air-free chemistry procedures to synthesize and separate iron oxide magnetic nanoparticles and subsequently modify the nanoparticle surface by using a chemical stripping agent. The morphology and chemical composition of the nanoparticles are characterized using electron microscopy and dynamic light scattering measurements. Additionally, magnetic characterization of the particles is performed using an inexpensive open-source (3D-printed) magnetophotometer. Possible modifications to the synthesis procedure, including the incorporation of dopants to modify the magnetic response and alternative characterization techniques, are discussed. The three-day synthesis, purification, and characterization laboratory will prepare students with crucial skills for advanced technology industries such as semiconductor manufacturing, nanomedicine, and green chemistry.

## Introduction and Background

In 2000, the National Nanotechnology Initiative was launched in the United States with four objectives designed to advance workforce training and technology development.^1^ Since then, the research community has made significant discoveries in nanotechnology research (first objective). However, the development and sustainment of educational resources to support the creation of a skilled workforce (third objective) have not received similar attention.^2^ As discussed in several longitudinal studies dating back to the mid-2000s, many undergraduate and graduate chemistry and chemical engineering laboratory experiments have not been updated to include recent technical advances and modern instrumentation.^3−5^ In addition to decreasing the utility of degrees, this has also resulted in increased attrition from science, technology, engineering, and mathematics (STEM) degree programs.^6^ In this context, the development of nanomaterial synthesis laboratories provides a clear strategy to increase engagement and retention in a wide range of degree programs.^7^

There are several examples of inquiry-based undergraduate teaching laboratories exploring fundamental nanoengineering concepts^8−11^ and training students in various nanocharacterization techniques.^12,13^ One family of nanomaterials commonly incorporated into teaching laboratories is optically responsive nanoparticles, including gold nanoparticles^14−18^ and fluorescent quantum dots.^11,19,20^ While these materials serve as an ideal platform for exploring complex quantum concepts, such as the discretization of energy states^21,22^ and manipulation of light at the nanoscale,^23,24^ the notable properties of these nanoparticles are limited to geometry and optical response. In contrast, nanomaterials can demonstrate a plethora of behaviors, including mechanical,^25^ optical,^2,23,24^ electrical,^26^ chemical,^27^ and more.^28−31^ One unique nanomaterial is iron oxide nanoparticles. Unlike quantum dots or metal nanoparticles, iron oxide nanoparticles exhibit size- and shape-tunable magnetic behavior.^27,32−34^ As a result of their magnetic response and low biotoxicity, iron oxide nanoparticles show promise in a wide range of applications and fields, including biomedical devices and imaging, energy storage and generation, and chemical processing.^28,31,35−37^ Thus, given their synergistic relationship to existing undergraduate nanoparticle laboratories and their emerging real-world applications, the synthesis and characterization of iron oxide nanoparticles provide a rich foundation for integration into an academic laboratory course.^38^

**Figure 1:**
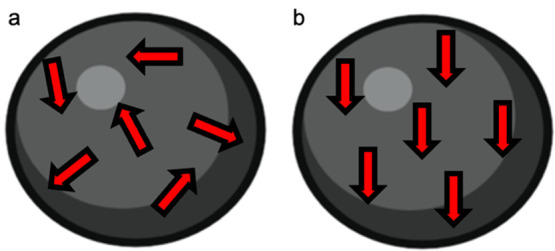
A paramagnetic nanoparticle (a) before and (b) during exposure to an external magnetic field. This figure was prepared by using BioRender.

Iron oxide nanoparticles fall into a larger class of materials known as paramagnetic materials. Unlike many commonly available magnetic materials, which rely on collective, long-range magnetic order to achieve an intrinsic magnetic response, paramagnetic and diamagnetic materials are considered nonmagnetic in the absence of an external magnetic field.^39^ However, in the presence of a field, their magnetic domains align in a single direction (Figure 1), and the particle exhibits a magnetic response. The electron pairing determines whether a material is diamagnetic or paramagnetic, which governs the sign of the magnetic susceptibility. Symmetric or spherical iron oxide nanoparticles demonstrate a paramagnetic response due to their unpaired electrons, and they have a positive magnetic susceptibility.^33^ This response is governed by the material composition, crystallographic uniformity, and size. Indirectly, it is also controlled by the sample preparation, as clusters of particles behave differently from well-dispersed particles. Given the diversity of applications for paramagnetic iron oxide nanoparticles, it is useful to measure their size, composition, and magnetic response before pursuing an application.^40−42^

In prior work, several methods have been used to characterize these properties in iron oxide nanoparticles specifically. The methods and their generated data are presented in Table 1. As can be observed, there are several options for measuring each parameter, depending on the availability of instrumentation. Additionally, depending on the method(s) selected, the results will be provided at either the single particle level or the population level. This provides a unique opportunity to compare the difference between single-particle and population-level data.

**Table 1:** Parameters of Interest and Characterization Methods

Parameter Measured	Measurement Method	Data Visualization	Type of Data
Particle size	Dynamic light scattering (DLS)^43^	Histogram	Population level
Particle size	Transmission electron microscopy (TEM)^44^	Image	Particle level
Particle size	Scanning electron microscopy (SEM)^45^	Image	Particle level
Particle size	Atomic force microscopy (AFM)^46^	2.5D image	Particle level
Dopant concentration	Energy dispersive X-ray spectroscopy (EDX/EDS)^47^	Spectra	Population level
Dopant concentration	X-ray diffractometry (XRD)^48^	Spectra	Population level
Magnetic response	Magnetophotometry (MAP)^49^	Graph	Population level
Magnetic response	SQUID^50^	Graph	Population level
Magnetic response	Magnetic resonance imaging (MRI)^51^	Image	Population level

## Experimental Details

**Figure 2:**
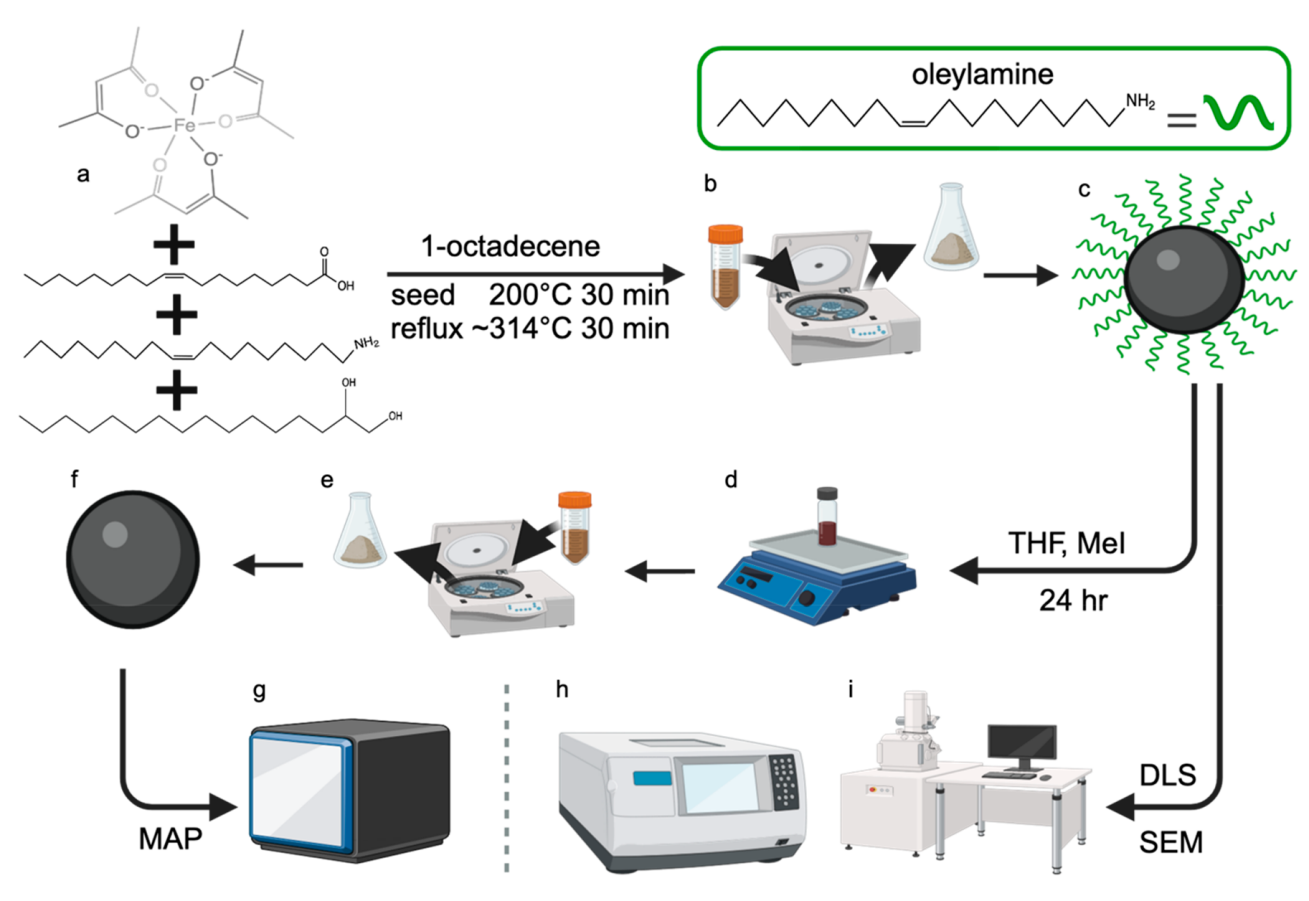
Overview of the full laboratory procedure. (a–c) Day 1 consists of nanoparticle synthesis, including (a) the Fe_3_O_4_ synthesis reaction, (b) isolating the iron oxide particles by centrifugation, and (c) drying the iron oxide nanoparticles coated with oleylamine ligands. (d–f) Day 2 includes (d) stripping the nanoparticles by iodomethane (MeI), (e) centrifugation to isolate the stripped iron oxide nanoparticles, and (f) drying the stripped Fe_3_O_4_ nanoparticles. (g–i) Characterization techniques used in day 3 include (g) magnetophotometry (MAP) using stripped nanoparticles, (h) scanning electron microscopy (SEM) using oleylamine-coated nanoparticles, and (i) dynamic light scattering (DLS) using oleylamine-coated nanoparticles. This figure was prepared by using BioRender.

The laboratory protocol is broken into three days: nanoparticle synthesis on day 1, sample preparation and nanoparticle surface treatment on day 2, and nanoparticle characterization on day 3. A general overview is illustrated in Figure 2, and the detailed protocol is contained in the supplementary Instructor and Student Manuals.

Single-crystal metal nanoparticles formed via a two-part synthesis of nucleation and growth steps are well understood to produce monodisperse size distributions with strong shape control.^52−56^ The one-pot two-part synthesis used here follows a previously published procedure with slight modifications to align with undergraduate laboratory expectations.^57,58^ Briefly, solid and liquid reagents are combined with the reaction solvent in an air-free environment (Figure 2a).^59^ Holding the reagents at an initial temperature of 200 °C cleaves the acetylacetone bonds in Fe(acac)_3_, allowing for nucleation of iron oxide sites. Further increasing the temperature until reflux, when particle growth is thermodynamically preferential, for a predetermined time leads to single-crystal and highly monodisperse particles (Figure 2b).

After purification, a subset of particles is further treated with iodomethane (MeI) to remove or “strip” the oleylamine ligands attached to the particle surface (Figure 2c). Samples are then prepared with the untreated and stripped particles (Figure 2d–f) to conduct size, shape, and magnetic response characterizations (Figure 2g–i). This student cohort used dynamic light scattering (DLS), scanning electron microscopy (SEM), and an open-source magnetophotometer (MAP)^49^ to analyze the particle size, shape, and magnetic response. Possible variations to the synthesis and alternative characterization methods are listed in Table 1 and are discussed in more detail in the Instructor Manual.

## Hazards and Safety

A concrete understanding of laboratory safety is essential for the pursuit of modern careers in science and engineering. This laboratory procedure involves the use of numerous hazardous chemicals and requires the use of appropriate mitigation measures. Before the start of the laboratory, all students received general laboratory safety training, which included instruction in proper handling of chemicals, personal protective equipment (PPE), mitigation measures (location and use of eye-wash stations, chemical showers, and fire extinguishers), and identification of chemicals by hazard type. During the laboratory, proper PPE (high-temperature lab coat, goggles, and nitrile or heat-resistant gloves) was used in accordance with standard chemistry practices. All chemistry procedures were conducted in a fume hood to limit airborne exposure, and a trained laboratory assistant was present throughout the procedure to assist when necessary. Additional hazard information is included in the Instructor and Student Manuals.

## Results and Discussion

### Educational Cohort

A cohort of students from the University of Southern California Viterbi School of Engineering with a minimum requirement of at least one semester equivalent of general chemistry volunteered to complete the lab to increase the diversity of their undergraduate research experiences. The students ranged from first-year undergraduates through fourth-year undergraduates pursuing a range of degree programs. All of the students had completed general laboratory safety training.

Under the supervision of the laboratory assistant, the students worked in groups of 2–3 over the course of the three-day lab, and each day was typically 3–4 h. The experiments were designed to support inquiry-based learning by encouraging the students to discover the answers to a series of scientific questions each day and expand their technical capabilities. These learning objectives can be summarized as follows:

1. Calculating the amount of reagents required using stoichiometric principles.
2. Practicing commonly used chemistry skills, including centrifugation.
3. Introducing students to laboratory techniques related to air-sensitive chemistry, such as using a glovebox and Schlenk line.
4. Executing various sample preparation protocols.
5. Learning and practicing nanomaterial characterization techniques.
6. Applying theoretical knowledge of characterization techniques and assessing the characteristics of the synthesized nanoparticles.
7. Developing data visualization and scientific communication skills.

Because this laboratory introduced new techniques and skills, the students were instructed in the principles behind each piece of equipment and executed the protocol themselves, but they were still closely monitored by a laboratory assistant at each step of the procedure (objectives 1–3). After carrying out the synthesis, students prepared samples for characterization according to the instructions listed in the Student Manual (objectives 4 and 5). Students performed all data analyses for subsequent discussion (objectives 6 and 7).

To assess if these learning objectives were achieved, the students’ understanding of the experimental and scientific principles was evaluated throughout the three days using diagnostic assessment, including pretests, and formative assessment, including passive and active observational techniques. For example, throughout the experiments, the laboratory assistant asked questions to assess the students’ understanding. The pretests and solutions as well as example questions are included in the supplementary documentation (see the Student Assessment and Student Assessment solutions documents).

Immediately following the conclusion of the laboratory, a summative assessment was performed, which included both qualitative and quantitative components. Notably, 100% of the students felt that the lab experiment should be integrated as a required element into the undergraduate curriculum, and 100% of the students felt that they gained valuable skills and/or knowledge applicable outside of this lab session. This strong positive response is notable given the students’ diverse academic backgrounds. However, the cohort had a mixed response when asked about the level of rigor of the lab. Given the wide range of course preparation and academic levels, this response is not surprising. To address this possible shortcoming, several variations are included in the Instructor Manual to increase the complexity.

In addition, a secondary reflective assessment was performed six months later. Upon reflection, the primary skills and knowledge imparted are the new data analysis methods and visualization techniques and the instrumentation methods. The students indicated the value of these as they relate to their ongoing coursework and their intended future career paths. It is notable that this group of students has already found value in this new knowledge and anticipate or are already applying it across a diverse range of fields, spanning from machine learning to therapeutics and biomedical engineering. This rapid impact highlights the relevance of this lab to an undergraduate engineering degree program. The entire assessment is included in the Instructor Manual.

### Nanoparticle Characterization

**Figure 3:**
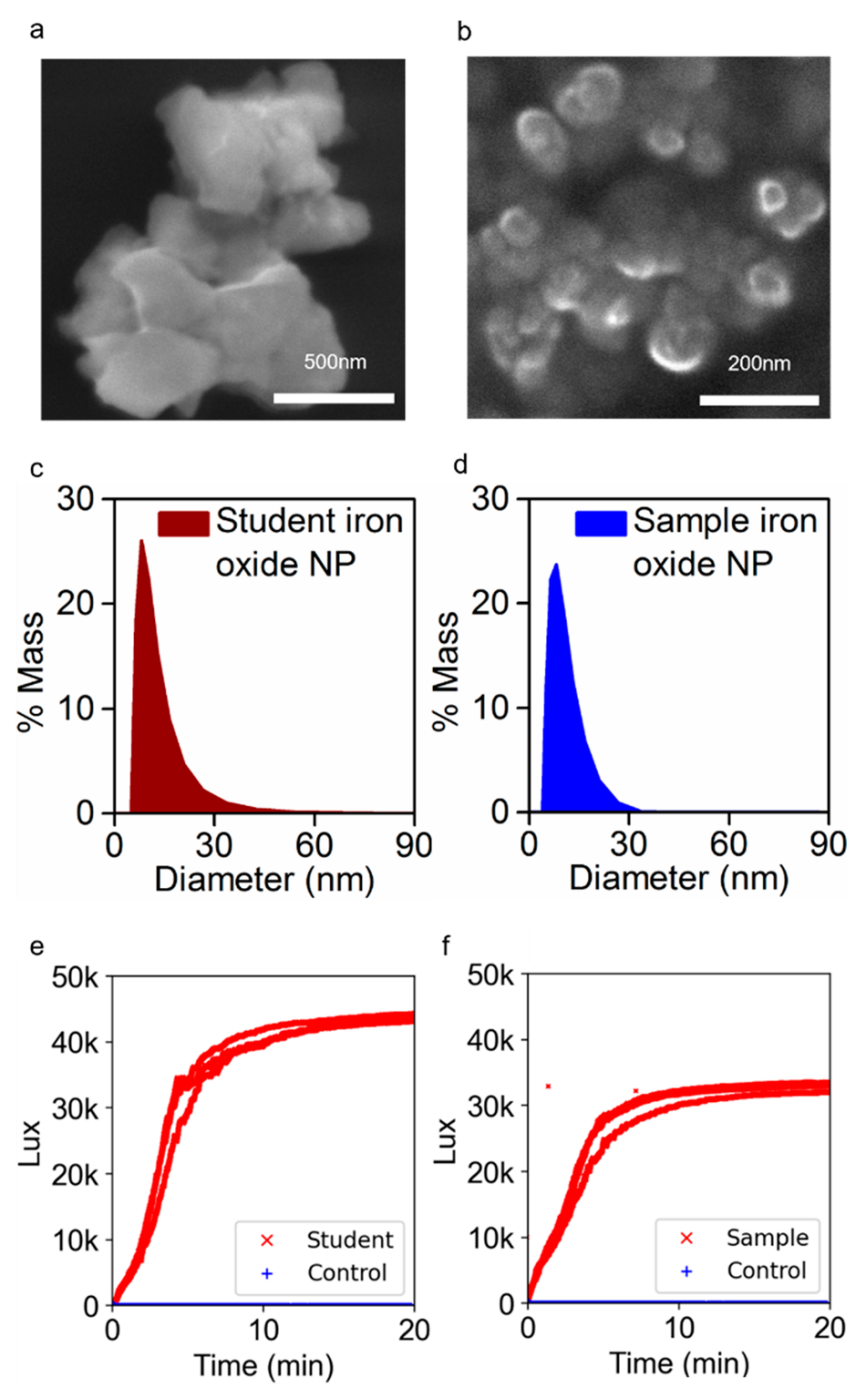
Iron oxide nanoparticle (a, b) SEM image, (c, d) DLS data, and (e, f) magnetic response data from the MAP system and ethanol control. Data taken with particles made by the student volunteers following the procedure are shown in panels (a), (c), and (e). Data taken with particles made by graduate assistant are shown in panels (b), (d), and (f).

To confirm the suitability of the synthetic protocols and the proposed characterization methods for an undergraduate laboratory setting, Figure 3 presents a series of results from nanoparticles prepared by the laboratory assistant and nanoparticles prepared by a single student group. Both sets of particles are synthesized following the procedure included in the Student Manual. Due to limitations in equipment access, the laboratory assistant performed the DLS measurements, but the students took SEM images and performed magnetic response testing with a MAP themselves. The students performed all sample preparation and data analysis. For all data sets in Figure 3, the results from the student group agreed with those from the laboratory assistant, demonstrating the robustness of the synthetic method.

The combination of SEM and DLS data provides an opportunity to analyze particle size at both the single particle level and the ensemble level. Based on published work by Sun et al., we anticipate the particle size to be between 10 and 15 nm.^57^ Each group prepared samples and acquired SEM images of their own batch of nanoparticles, as seen in Figure 3a. Notably, the image shows particle clumps in the 100 nm range, and individual particles are not clearly resolved (Figure 3a). For the particles synthesized by the laboratory assistant, the diameter determined from SEM images is in the 15 nm range (Figure 3b). The DLS data reveal similar particle sizes (diameters of 12 nm) for both the student and laboratory assistant samples (Figure 3c,d). Lastly, students confirmed the paramagnetic response of the particles using a custom MAP system (Figure 3e,f). As can be observed in Figure 3e,f, the particles synthesized by either the students or the laboratory assistant exhibit a clear positive magnetic response when in the presence of a permanent magnetic field, indicating a positive magnetic susceptibility value.

## Summary and Conclusion

In summary, we have detailed a research-based three-day advanced undergraduate laboratory protocol that includes the synthesis of iron oxide magnetic nanoparticles, modification of the surface coating, and characterization of the particle properties. The experiments can be easily modified based on available equipment. They are designed to combine principles from chemistry, materials science, and physics to expose students to interdisciplinary research and modern instrumentation while utilizing inexpensive reagents and commonly accessible characterization techniques. They also serve as a bridge connecting seemingly disparate coursework. While this three-day laboratory experiment was piloted with a cohort of undergraduate students, it could also be suitable for a graduate student experimental methods course.

## Supporting Information Available

Instructor Manual: detailed protocols, trouble-shooting suggestions, material and equipment tables, and educational assessment (PDF, DOCX)
Student Manual: step-by-step protocols and material and equipment tables (PDF, DOCX)
Student Assessment: suggested prelaboratory exercises to enrich student learning (PDF, DOCX)
Student Assessment solutions (PDF, DOCX)

## References

[ref1] National Academies of Sciences, Engineering, and Medicine; Division on Engineering and Physical Sciences; National Materials and Manufacturing Board; Committee on National Nanotechnology Initiative: A Quadrennial Review. In A Quadrennial Review of the National Nanotechnology Initiative: Nanoscience, Applications, and Commercialization; The National Academies Press, Washington, D.C., U.S., 2020. 32931182

[ref2] Gottfried D. S. Review of Nanotechnology in Undergraduate Education. J. Chem. Educ. 2011, 88 (5), 544–545. 10.1021/ed2001126.

[ref3] Sheppard S. D.; Macatangay K.; Colby A.; Sullivan W. M. In Educating Engineers: Designing for the Future of the Field. Book Highlights; Carnegie Foundation for the Advancement of Teaching, 2008. https://eric.ed.gov/?id=ED504076 (accessed 2023-08-10).

[ref4] National Academies of Sciences, Engineering, and Medicine; Division on Earth and Life Studies; Division of Behavioral and Social Sciences and Education; Committee for Convocation on Integrating Discovery-Based Research into the Undergraduate Curriculum. In Integrating Discovery-Based Research into the Undergraduate Curriculum: Report of a Convocation; The National Academies Press, Washington, D.C., U.S., 2015. 10.17226/21851.

[ref5] National Academy of Engineering; National Academies of Sciences, Engineering, and Medicine; Division on Earth and Life Studies; Board on Chemical Sciences and Technology; Committee on Chemical Engineering in the 21st Century: Challenges and Opportunities. In New Directions for Chemical Engineering; The National Academies Press, Washington, D.C., 2022. 10.17226/26342.

[ref6] Hallinen J. STEM. Encyclopedia Britannica; 2023. https://www.britannica.com/topic/STEM-education.

[ref7] Weaver G. C.; Russell C. B.; Wink D. J. Inquiry-Based and Research-Based Laboratory Pedagogies in Undergraduate Science. Nat. Chem. Biol. 2008, 4 (10), 577–580. 10.1038/nchembio1008-577. 18800041

[ref8] Bentley A. K.; Skrabalak S. E. A Primer on Lattice Planes, Crystal Facets, and Nanoparticle Shape Control. J. Chem. Educ. 2023, 100 (9), 3425–3433. 10.1021/acs.jchemed.3c00371.

[ref9] Nanda S.; Nanda K. K. Identifying the Accuracy of Various Approaches for Determining the Fraction of Surface Atoms in a Nanoparticle to Deepen Students’ Understanding of Size-Dependent Properties. J. Chem. Educ. 2021, 98 (6), 1982–1987. 10.1021/acs.jchemed.0c01247.

[ref10] Vinnacombe-Willson G. A.; Chiang N.; Weiss P. S.; Tolbert S. H.; Scarabelli L. Seeded-Growth Experiment Demonstrating Size- and Shape-Dependence on Gold Nanoparticle–Light Interactions. J. Chem. Educ. 2021, 98 (2), 546–552. 10.1021/acs.jchemed.0c01150. 34024937 PMC8133700

[ref11] Nordell K. J.; Boatman E. M.; Lisensky G. C. A Safer, Easier, Faster Synthesis for CdSe Quantum Dot Nanocrystals. J. Chem. Educ. 2005, 82 (11), 1697 10.1021/ed082p1697.

[ref12] Lam M.; Schwarz C.; Sharma R.; Donnelly J. An Introduction to Scanning Electron Microscopy and Science Communication Skills for Undergraduate Chemistry Students. J. Chem. Educ. 2023, 100 (7), 2802–2808. 10.1021/acs.jchemed.3c00076.

[ref13] Vahedi A.; Farnoud A. M. Novel Experimental Modules To Introduce Students to Nanoparticle Characterization in a Chemical Engineering Course. J. Chem. Educ. 2019, 96 (9), 2029–2035. 10.1021/acs.jchemed.9b00423. 34045773 PMC8153379

[ref14] Aftenieva O.; Schletz D.; Meyer A.; Kühne T.; Schmalzriedt S.; Niethammer M.; König T. A. F. Development of a Teaching Platform about Plasmonics Based on the Color Perception of Colloidal Gold. J. Chem. Educ. 2021, 98 (8), 2566–2573. 10.1021/acs.jchemed.1c00183.

[ref15] Cionti C.; Stucchi M.; Meroni D. Mimicking Stained Glass: A Hands-On Activity for the Preparation and Characterization of Silica Films Colored with Noble Metal Ions and Nanoparticles. J. Chem. Educ. 2022, 99 (3), 1516–1522. 10.1021/acs.jchemed.1c01141.

[ref16] Jenkins J. A.; Wax T. J.; Zhao J. Seed-Mediated Synthesis of Gold Nanoparticles of Controlled Sizes To Demonstrate the Impact of Size on Optical Properties. J. Chem. Educ. 2017, 94 (8), 1090–1093. 10.1021/acs.jchemed.6b00941.

[ref17] Larm N. E.; Essner J. B.; Thon J. A.; Bhawawet N.; Adhikari L.; St Angelo S. K.; Baker G. A. Single Laboratory Experiment Integrating the Synthesis, Optical Characterization, and Nanocatalytic Assessment of Gold Nanoparticles. J. Chem. Educ. 2020, 97 (5), 1454–1459. 10.1021/acs.jchemed.9b00819.

[ref18] Quinson J. Room Temperature Surfactant-Free Syntheses of Gold Nanoparticles in Alkaline Mixtures of Water and Alcohols: A Model System to Introduce Nanotechnology and Green Chemistry to Future Chemists and Engineers. J. Chem. Educ. 2023, 100 (9), 3612–3619. 10.1021/acs.jchemed.3c00492.

[ref19] Lisensky G.; McFarland-Porter R.; Paquin W.; Liu K. Synthesis and Analysis of Zinc Copper Indium Sulfide Quantum Dot Nanoparticles. J. Chem. Educ. 2020, 97 (3), 806–812. 10.1021/acs.jchemed.9b00642.

[ref20] Dhawan G.; Singh I.; Dhawan U.; Kumar P. Synthesis and Characterization of Nanoselenium: A Step-by-Step Guide for Undergraduate Students. J. Chem. Educ. 2021, 98 (9), 2982–2989. 10.1021/acs.jchemed.0c01467.

[ref21] Winkler L. D.; Arceo J. F.; Hughes W. C.; DeGraff B. A.; Augustine B. H. Quantum Dots: An Experiment for Physical or Materials Chemistry. J. Chem. Educ. 2005, 82 (11), 1700 10.1021/ed082p1700.

[ref22] Winkelmann K.; Noviello T.; Brooks S. Preparation of CdS Nanoparticles by First-Year Undergraduates. J. Chem. Educ. 2007, 84 (4), 709 10.1021/ed084p709.

[ref23] Halas N. J.; Lal S.; Chang W.-S.; Link S.; Nordlander P. Plasmons in Strongly Coupled Metallic Nanostructures. Chem. Rev. 2011, 111 (6), 3913–3961. 10.1021/cr200061k. 21542636

[ref24] Hartland G. V. Optical Studies of Dynamics in Noble Metal Nanostructures. Chem. Rev. 2011, 111 (6), 3858–3887. 10.1021/cr1002547. 21434614

[ref25] Ramos M.; Ortiz-Jordan L.; Hurtado-Macias A.; Flores S.; Elizalde-Galindo J. T.; Rocha C.; Torres B.; Zarei-Chaleshtori M.; Chianelli R. R. Hardness and Elastic Modulus on Six-Fold Symmetry Gold Nanoparticles. Materials 2013, 6 (1), 198–205. 10.3390/ma6010198. 28809302 PMC5452105

[ref26] Fischer J. E.; Johnson A. T. Electronic Properties of Carbon Nanotubes. Curr. Opin. Solid State Mater. Sci. 1999, 4 (1), 28–33. 10.1016/S1359-0286(99)80007-2.

[ref27] Wu L.; Mendoza-Garcia A.; Li Q.; Sun S. Organic Phase Syntheses of Magnetic Nanoparticles and Their Applications. Chem. Rev. 2016, 116 (18), 10473–10512. 10.1021/acs.chemrev.5b00687. 27355413

[ref28] Tanaka S.; Kaneti Y. V.; Septiani N. L. W.; Dou S. X.; Bando Y.; Hossain Md. S. A.; Kim J.; Yamauchi Y. A Review on Iron Oxide-Based Nanoarchitectures for Biomedical, Energy Storage, and Environmental Applications. Small Methods 2019, 3 (5), 1800512 10.1002/smtd.201800512.

[ref29] Baabu P. R. S.; Kumar H. K.; Gumpu M. B.; Babu K J.; Kulandaisamy A. J.; Rayappan J. B. B. Iron Oxide Nanoparticles: A Review on the Province of Its Compounds, Properties and Biological Applications. Materials 2023, 16 (1), 59 10.3390/ma16010059. PMC9820855 36614400

[ref30] Salih S. J.; Mahmood W. M. Review on Magnetic Spinel Ferrite (MFe2O4) Nanoparticles: From Synthesis to Application. Heliyon 2023, 9 (6), e16601 10.1016/j.heliyon.2023.e16601. 37274649 PMC10238938

[ref31] Lee N.; Yoo D.; Ling D.; Cho M. H.; Hyeon T.; Cheon J. Iron Oxide Based Nanoparticles for Multimodal Imaging and Magnetoresponsive Therapy. Chem. Rev. 2015, 115 (19), 10637–10689. 10.1021/acs.chemrev.5b00112. 26250431

[ref32] Delgado T.; Villard M. Spin Crossover Nanoparticles. J. Chem. Educ. 2022, 99 (2), 1026–1035. 10.1021/acs.jchemed.1c00990.

[ref33] Kim D. K.; Zhang Y.; Voit W.; Rao K. V.; Muhammed M. Synthesis and Characterization of Surfactant-Coated Superparamagnetic Monodispersed Iron Oxide Nanoparticles. J. Magn. Magn. Mater. 2001, 225 (1), 30–36. 10.1016/S0304-8853(00)01224-5.

[ref34] Chi Y.; Yuan Q.; Li Y.; Tu J.; Zhao L.; Li N.; Li X. Synthesis of Fe3O4@SiO2–Ag Magnetic Nanocomposite Based on Small-Sized and Highly Dispersed Silver Nanoparticles for Catalytic Reduction of 4-Nitrophenol. J. Colloid Interface Sci. 2012, 383 (1), 96–102. 10.1016/j.jcis.2012.06.027. 22789800

[ref35] Fruntke A.; Behnke M.; Stafast L. M.; Träder T.; Dietel E.; Vollrath A.; Weber C.; Schubert U. S.; Wilke T. Targeted Drug Delivery: Synthesis of Smart Nanocarriers for School Chemistry Education. J. Chem. Educ. 2023, 100 (2), 751–759. 10.1021/acs.jchemed.2c00422.

[ref36] Rattanakit P. Open Inquiry-Based Laboratory Project on Plant-Mediated Green Synthesis of Metal Nanoparticles and Their Potential Applications. J. Chem. Educ. 2021, 98 (12), 3984–3991. 10.1021/acs.jchemed.1c00300.

[ref37] Reddy L. H.; Arias J. L.; Nicolas J.; Couvreur P. Magnetic Nanoparticles: Design and Characterization, Toxicity and Biocompatibility, Pharmaceutical and Biomedical Applications. Chem. Rev. 2012, 112 (11), 5818–5878. 10.1021/cr300068p. 23043508

[ref38] Ahlburg J. V.; Menhinnitt Z.; Thomas-Hunt J.; Saura-Múzquiz M.; Christensen M. Synthesis and Characterization of a Magnetic Ceramic Using an Easily Accessible Scale Setup. J. Chem. Educ. 2021, 98 (8), 2632–2637. 10.1021/acs.jchemed.1c00143.

[ref39] Young H. D.; Freedman R. A.; Ford A. L. In Sears and Zemansky’s University Physics : With Modern Physics, 12th ed.; Pearson Addison-Wesley, San Francisco, California, U.S., 2008.

[ref40] Chi Y.; Yuan Q.; Li Y.; Tu J.; Zhao L.; Li N.; Li X. Synthesis of Fe3O4@SiO2–Ag Magnetic Nanocomposite Based on Small-Sized and Highly Dispersed Silver Nanoparticles for Catalytic Reduction of 4-Nitrophenol. J. Colloid Interface Sci. 2012, 383 (1), 96–102. 10.1016/j.jcis.2012.06.027. 22789800

[ref41] Sun W.; Li Q.; Gao S.; Shang J. K. Monometallic Pd/Fe3O4 Catalyst for Denitrification of Water. Applied Catalysis B: Environmental 2012, 125, 1–9. 10.1016/j.apcatb.2012.05.014.

[ref42] Tan H.; Xue J. M.; Shuter B.; Li X.; Wang J. Synthesis of PEOlated Fe _3_ O _4_ @SiO _2_ Nanoparticles via Bioinspired Silification for Magnetic Resonance Imaging. Adv. Funct Materials 2010, 20 (5), 722–731. 10.1002/adfm.200901820.

[ref43] Biganzoli D.; Ferri F. Statistical Analysis of Dynamic Light Scattering Data: Revisiting and beyond the Schätzel Formulas. Opt. Express, OE 2018, 26 (22), 29375–29392. 10.1364/OE.26.029375. 30470102

[ref44] Transmission Electron Microscopy. Nanoscience Instruments. https://www.nanoscience.com/techniques/transmission-electron-microscopy/ (accessed 2024-01-30).

[ref45] Scanning Electron Microscopy. Nanoscience Instruments. https://www.nanoscience.com/techniques/scanning-electron-microscopy/ (accessed 2024-01-30).

[ref46] Atomic Force Microscopy. Nanoscience Instruments. https://www.nanoscience.com/techniques/atomic-force-microscopy/ (accessed 2024-01-30).

[ref47] Materials Science form - US. https://www.thermofisher.com/us/en/reference-components/MSD-reference-components/instruments-reference-components/materials-science-form.html (accessed 2024-01-30).

[ref48] Holder C. F.; Schaak R. E. Tutorial on Powder X-Ray Diffraction for Characterizing Nanoscale Materials. ACS Nano 2019, 13 (7), 7359–7365. 10.1021/acsnano.9b05157. 31336433

[ref49] Scholtz A.; Paulson J.; Nunez V.; Armani A. M. Open-Source Magnetophotometer (MAP) for Nanoparticle Characterization. arXiv 2023, 10.48550/arXiv.2401.01903.

[ref50] Vettoliere A.; Silvestrini P.; Granata C. 3 - Superconducting Quantum Magnetic Sensing. In Quantum Materials, Devices, and Applications; Henini M., Rodrigues M. O., Eds.; Elsevier, 2023; p 43–85. 10.1016/B978-0-12-820566-2.00001-6.

[ref51] Estelrich J.; Sánchez-Martín M. J.; Busquets M. A. Nanoparticles in Magnetic Resonance Imaging: From Simple to Dual Contrast Agents. Int. J. Nanomed. 2015, 10, 1727–1741. 10.2147/IJN.S76501. PMC4358688 25834422

[ref52] Xia Y.; Gilroy K. D.; Peng H.-C.; Xia X. Seed-Mediated Growth of Colloidal Metal Nanocrystals. Angew. Chem., Int. Ed. 2017, 56 (1), 60–95. 10.1002/anie.201604731. 27966807

[ref53] Tao A. R.; Habas S.; Yang P. Shape Control of Colloidal Metal Nanocrystals. Small 2008, 4 (3), 310–325. 10.1002/smll.200701295.

[ref54] Sun W.; Li Q.; Gao S.; Shang J. K. Monometallic Pd/Fe3O4 Catalyst for Denitrification of Water. Applied Catalysis B: Environmental 2012, 125, 1–9. 10.1016/j.apcatb.2012.05.014.

[ref55] Skrabalak S. E. Symmetry in Seeded Metal Nanocrystal Growth. Acc. Mater. Res. 2021, 2 (8), 621–629. 10.1021/accountsmr.1c00077.

[ref56] Personick M. L.; Mirkin C. A. Making Sense of the Mayhem behind Shape Control in the Synthesis of Gold Nanoparticles. J. Am. Chem. Soc. 2013, 135 (49), 18238–18247. 10.1021/ja408645b. 24283259

[ref57] Sun S.; Zeng H.; Robinson D. B.; Raoux S.; Rice P. M.; Wang S. X.; Li G. Monodisperse MFe _2_ O _4_ (M = Fe, Co, Mn) Nanoparticles. J. Am. Chem. Soc. 2004, 126 (1), 273–279. 10.1021/ja0380852. 14709092

[ref58] Ren Y.; Li Y.; Xu N.; Guo K.; Xu Z.; Chen X.; Liu H.; Gao J. Regulation of Saturation Magnetization of Magnetite by Doping with Group III Elements. Phys. Chem. Chem. Phys. 2023, 25 (48), 33152–33158. 10.1039/D3CP03789D. 38047897

[ref59] Borys A. M. An Illustrated Guide to Schlenk Line Techniques. Organometallics 2023, 42 (3), 182–196. 10.1021/acs.organomet.2c00535.

