# Supplementary material for: Synthesis and Characterization of Superparamagnetic Iron Oxide Nanoparticles: A Series of Laboratory Experiments
Source: J Chem Educ. 2024 Apr 9;101(5):2039–44. doi: 10.1021/acs.jchemed.3c00996 (PMC11097384; doi:10.1021/acs.jchemed.3c00996)
Supplement: Supplementary file 2 — ed3c00996_si_002.docx [file ed3c00996_si_002.docx]

Supporting Information for:

**Synthesis and Characterization of Superparamagnetic**

**Nanoparticles: A Series of Laboratory Experiments**

Armando D. Urbina^1+^, Hari Sridhara^1+^, Alexis Scholtz^2+^, Andrea M. Armani^1,2,3^*

^1^ Mork Family Department of Chemical Engineering and Materials Science, University of Southern California, Los Angeles, CA 90089, USA

^2^ Alfred E. Mann Department of Biomedical Engineering, University of Southern California, Los Angeles, CA 90089, USA

^3^ Ellison Institute of Technology, Los Angeles, CA 90064, USA

^+^ These authors contributed equally.

[*aarmani@eit.org](mailto:*aarmani@eit.org)

**Synthesis and Characterization of Superparamagnetic**

**Iron Oxide Nanoparticles**

Instructor’s Manual

Table of Contents

[Table of Contents 2](#_Toc158440395)

[preface 4](#_Toc158440396)

[Background 5](#_Toc158440397)

[Overview of Experiment 7](#_Toc158440398)

[Full List of Materials and Equipment 8](#_Toc158440399)

[Chemical Reagents 10](#_Toc158440400)

[Hazards 12](#_Toc158440401)

[Day 1: Magnetic Nanoparticle Synthesis 13](#_Toc158440402)

[Day 1 Overview 13](#_Toc158440403)

[Day 1 Equipment, Materials, and Chemicals 14](#_Toc158440404)

[Day 1 Synthesis Protocol 16](#_Toc158440405)

[Day 2: Ligand Stripping and Sample Preparation 22](#_Toc158440406)

[Day 2 Overview 22](#_Toc158440407)

[Day 2 Equipment, Materials, and Chemicals 23](#_Toc158440408)

[Day 2: Ligand Stripping and Sample Preparation Protocol 24](#_Toc158440409)

[Day 3: Nanoparticle Characterization and Data Analysis 26](#_Toc158440410)

[Day 3 Overview 26](#_Toc158440411)

[Day 3 Equipment, Materials, and Chemicals 27](#_Toc158440412)

[Day 3 Characterization Protocol 28](#_Toc158440413)

[Possible Variations 30](#_Toc158440414)

[Nanoparticle Synthesis 30](#_Toc158440415)

[Characterization 31](#_Toc158440416)

[Data Analysis 31](#_Toc158440417)

[Educational Assessment 32](#_Toc158440418)

[Student Volunteer Background 32](#_Toc158440419)

[Assessment Strategy 32](#_Toc158440420)

[Pre-laboratory Assessment 33](#_Toc158440421)

[Formative Assessment 33](#_Toc158440422)

[Summative Assessment 34](#_Toc158440423)

[Reflective Assessment 36](#_Toc158440424)

[References 40](#_Toc158440425)

preface

While there is a need for overlap between the Instructor and Student Manuals to provide motivation, relevant background, and instructions, we wanted to highlight the differences between the two documents. Shared sections between documents, such as background and hazards, have been tailored to the relevant audience (i.e. instructor vs. student). The Instructor Manual contains additional details and practical notes for the instructor which were learned during our experiences with students. Likewise, the Student Manual provides additional instructions and space for students to record relevant quantities and note observations that will be necessary to complete their post-assessment.

Background

Nanomaterials have recently helped advance electronics, medicine, and manufacturing technologies. Thus, it is important for chemical engineering and materials science curricula to prepare students for these careers through exposure to nanomaterials and training on relevant skills and techniques. Iron oxide nanoparticles are chosen for this teaching exercise both because of the novelty of this material in educational settings and their exceptional magnetic response, low biotoxicity, and ease of fabrication^1–4^ that make them so useful. Further, iron oxide nanoparticles have vast applications in technologies such as imaging contrast agents, catalysis, and data storage.^5–9^

These applications leverage the specific magnetic properties of iron oxide nanoparticles, which exhibit paramagnetism. Unlike many magnetic materials which rely on collective, long-range magnetic order to achieve an intrinsic magnetic response, paramagnetic and diamagnetic materials are considered nonmagnetic in the absence of an external magnetic field. However, in the presence of a field, their magnetic domains align in a single direction, as seen in Figure S1, and the particle exhibits a magnetic response. The electron pairing determines if a material is diamagnetic or paramagnetic, which governs the sign of the magnetic susceptibility. Symmetric or spherical iron oxide nanoparticles demonstrate their paramagnetic response due to their unpaired electrons and have a positive magnetic susceptibility. If the particles are small enough, they may exhibit superparamagnetism, which is when particles exhibit very short magnetic relaxation response times, resulting in superior paramagnetic properties.

**
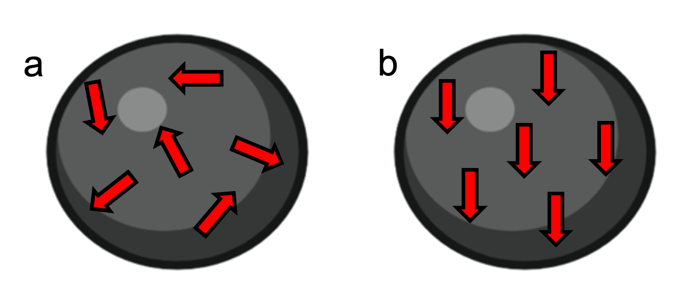
**

Figure S1. Paramagnetic nanoparticle (a) before and (b) during exposure to an external magnetic field pointing towards the bottom of the page.

Given the diversity of applications for paramagnetic iron oxide nanoparticles, it is useful to measure their size, composition, and magnetic response.

As part of this series of experiments, the following skills will be developed:

- Air-sensitive chemistry (ex. Schlenk line, glove box)
- Laboratory techniques (ex. centrifuge, sonicator)
- Synthesis equipment proficiency (ex. heating mantle, condenser)
- Characterization techniques (ex. DLS, SEM, magnetophotometry)
- Data analysis methods (ex. linear regression, distribution, size statistics)

Overview of Experiment

The purpose of this series of laboratories is to demonstrate how the superparamagnetic behavior of iron oxide nanoparticles depends on the synthesis conditions and material doping. Day 1 focuses on the synthesis (Figure S2a-c), Day 2 on the surface treatment and sample preparation of the nanoparticles (Figure S2d-f), and Day 3 on the nanoparticle characterization and analysis (Figure S2g-i). This protocol and corresponding materials list are based on the suggested batch size of 80 mL. Full lists of materials, equipment, and chemicals needed are compiled in Sections 3 and 4.


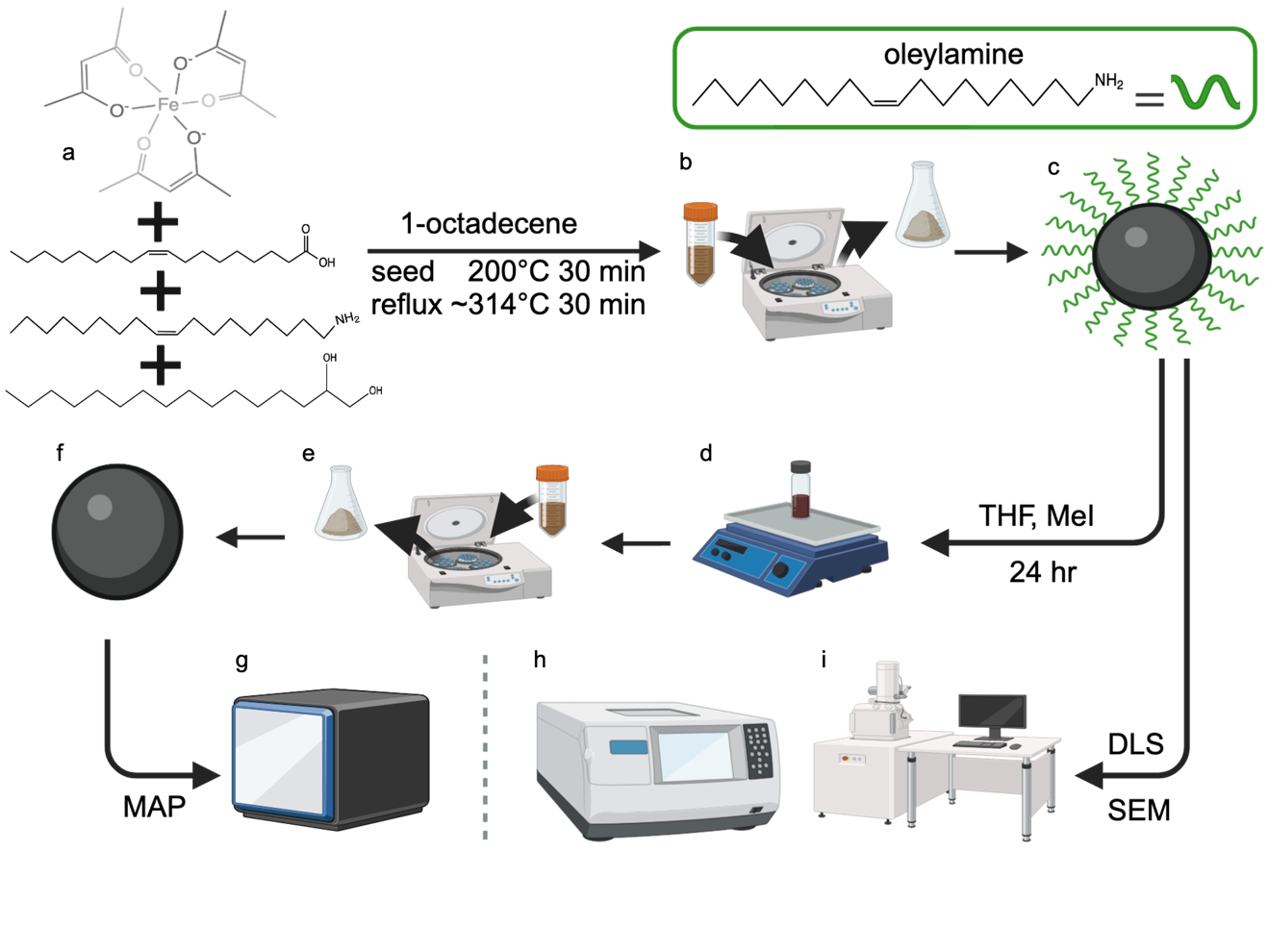


Figure S2. Overview of the full laboratory procedure. (a-c) Day 1 consists of nanoparticle synthesis including (a) the Fe_3_O_4_ synthesis reaction, (b) isolating the iron oxide particles by centrifuge, and (c) drying the iron oxide nanoparticles coated with oleylamine ligands. (d-f) Day 2 includes (d) the stripping of nanoparticles by iodomethane (MeI), (e) centrifugation to isolate the stripped iron oxide nanoparticles, and (f) drying the stripped Fe_3_O_4_ nanoparticles. (g-i) Characterization techniques used in Day 3 include (g) magnetophotometry (MAP) using stripped nanoparticles, (h) Scanning Electron Microscopy (SEM) using oleylamine-coated nanoparticles, and (i) Dynamic Light Scattering (DLS) using oleylamine-coated nanoparticles. This figure was prepared using BioRender.

Full List of Materials and Equipment

Full lists of materials and equipment are found in Tables S1 and S2. Lists of material and equipment required for each individual lab session can be found in the Materials and Equipment section for each day.

Note that the listed sizes for glassware and other measurement tools assume the 80 mL batch size indicated in the Student Manual and Assessment but can easily be modified or substituted as needed. However, we do not recommend batch sizes lower than 50 mL as particle separation with smaller batches becomes challenging.

| Table S1. Compiled List of Equipment Required for the Full Procedure | |
| --- | --- |
| Equipment | Number Needed |
| Fume hood | 1 |
| Electronic balance with 0.01 g resolution | 1 |
| Schlenk line | 1 |
| Stir plate | 1 |
| Heating mantle | 1 |
| Inconel or stainless steel thermocouple | 1 |
| Thermocouple adapter | 1 |
| Centrifuge | 1 |
| Vacuum desiccator (optional) | 1 |
| Ring stand | 1 |
| Non-contact mixing equipment such as a shake table or inversion table | 1 |
| 100-1000µL pipette^a^ | 1 |
| Vortex mixer | 1 |
| Sonicator | 1 |
| ^a^Pipette will be used to measure 2 mL of dispersion for sample preparation. | |

| Table S2. Compiled List of Materials and Consumables for the Full Procedure | |
| --- | --- |
| Materials and Consumables | Number Needed |
| 250 mL three-neck round-bottom flask | 1 |
| Egg-shaped magnetic stir bar^a^ | 1 |
| Rubber septa | 2 |
| 5 mL syringe | 2 |
| Hypodermic needle | 1 |
| Condenser, pump, container, and tubing |  |
| Luer-lock needle | 1 |
| Glassware clips | 3 |
| Metal hose clamps | 3 |
| 100 mL graduated cylinder | 1 |
| Vacuum grease |  |
| 50 mL centrifuge tube | 4 |
| Neodymium block magnet^b^ | 1 |
| Disposable cuvettes with lid^c^ | 2 |
| Si wafer chip (1 cm x 1 cm) | 1 |
| 250 mL beaker | 1 |
| 50 mL glass vial | 1 |
| Disposable tips for 100-1000 µL pipette | ≥7 |
| 10 mL glass vial | 2 |
| ^a^The stir bar should be appropriately sized to provide sufficient mixing while maintaining a stable rotation.  ^b^This magnet can be the same one used in the magnetophotometer.  ^c^The material depends on the choice of solvent; we used PMMA cuvettes. Reusable glass cuvettes may also be used. | |

Chemical Reagents

A full list of reagents for the laboratory protocol is provided in Table S3; this assumes a batch size of 80 mL. The reagents listed in this protocol also have specific requirements for storage to maintain chemical stability as detailed in Table S4.

The smallest recommended minimum batch size is 50 mL, which should yield approximately 800 mg of particles. Based on our experience, it is not recommended to scale down the reaction below this threshold due to issues with particle separation. A minimum of 100 mg of dried particles are needed to complete the suggested analysis methods provided in this procedure. The reaction can be scaled up to batch sizes of 150 mL, but this will require longer heating times and appropriately scaled reagents and glassware.

| Table S3. Consumables Listed on a Per-Experiment Basis, Assuming a Batch Size of 80mL of Solvent | | | | | | | |
| --- | --- | --- | --- | --- | --- | --- | --- |
| Reagent | Shorthand | State | Purity^a^ | Vendor^a^ | Container Size | Quantity Needed per Experiment | Experiments per Container |
| Iron (III) acetylacetonate | Fe(acac)_3_ | Solid | ≥98.0% | TCI | 25 g | 2.82 g | 8 |
| Cobalt (II) acetylacetonate^b^ | Co(acac)_2_ | Solid | ≥97.0% | TCI | 25 g | 1.03 g | 24 |
| Manganese (II) acetylacetonate^b^ | Mn(acac)_2_ | Solid | ≥98.0% | TCI | 25 g | 1.03 g | 24 |
| 1,2-hexadecanediol |  | Solid | ≥98.0% | TCI | 25 g | 5.17 g | 4 |
| Oleic acid |  | Liquid | 50% | TCI | 25 mL | 7.57 mL | 3 |
| Oleylamine |  | Liquid | ≥85.0% | TCI | 25 mL | 7.90 mL | 3 |
| 1-octadecene |  | Liquid | 90% | Sigma-Aldrich | 250 mL | 80 mL | 3 |
| Reagent alcohol |  | Liquid | 94-96% | VWR | 4L | ~500mL | ~8 |
| Hexane (mixture of isomers) |  | Liquid | >98.5% | VWR | 4L | ~500mL | ~8 |
| Hexane (HPLC grade) |  | Liquid | >97% | VWR | 1L | ~4mL | ~250 |
| Ethanol | EtOH | Liquid | 99.5% | VWR | 1 gal | ~6 mL | ~630 |
| Iodomethane | MeI | Liquid | ≥99% | BeanTown Chemical | 50 g | 0.7 mL | 31 |
| Tetrahydrofuran | THF | Liquid | ≥99.8% | Sigma-Aldrich | 100 mL | 20 mL | 5 |
| ^a^The listed purities and vendors proved successful in our experimentation but are not mandatory for a successful experiment.  ^b^Note the use of Co(acac)_2_ and Mn(acac)_2_ as dopants is optional and is described as a potential modification in this document. | | | | | | | |

| Table S4. Reagent Sensitivity, Storage, and Stability Information | | | | |
| --- | --- | --- | --- | --- |
| Reagent | Shorthand | Sensitivity | Storage | Stability |
| Iron (III) acetylacetonate | Fe(acac)_3_ | None | Keep in a dry place | Chemically stable |
| Cobalt (II) acetylacetonate | Co(acac)_2_ | None | Keep in a dry place | Chemically stable |
| Manganese (II) acetylacetonate | Mn(acac)_2_ | None | Keep in a dry place | Chemically stable |
| 1,2-hexadecanediol |  | None | Keep in a dry place | Chemically stable |
| Oleic acid |  | Oxygen | Oxygen-free environment (glove box) | Chemically stable under recommended storage conditions |
| Oleylamine |  | Oxygen | Oxygen-free environment (glove box) | Chemically stable under recommended storage conditions |
| 1-octadecene |  | None | Keep in a dry place below 30 ºC | Chemically stable |
| Reagent alcohol |  | None | Keep in a well-ventilated place | Chemically stable |
| Hexane |  | None | Keep in a well-ventilated place | Chemically stable |
| Ethanol | EtOH | None | Keep in a well-ventilated place | Chemically stable |
| Iodomethane | MeI | Light | Keep in a dry place below 30 ºC | Volatile |
| Tetrahydrofuran | THF | None | Keep in a cool dry place | Flammable |

Hazards

A concrete understanding of laboratory safety is essential preparation for careers in science and engineering. This laboratory procedure involves use of numerous hazardous chemicals and requires the use of appropriate mitigation measures. Proper personal protective equipment (PPE), including a high-temperature lab coat, goggles, and nitrile or heat-resistant (when necessary) gloves, should be used in accordance with standard chemistry practices. Beyond standard safety practices and initial general laboratory safety training, this laboratory also involves more extensive work with powder, vacuum and Schlenk lines, and flammable solvents, all of which is detailed below. A trained laboratory assistant should be present throughout the procedure to supervise all steps and assist when necessary.

When working with powders, it is important for students to measure their powder reagents in a well-ventilated area to avoid accidental inhalation. It is preferable to have an exhaust snorkel above the electronic scale which will aspirate any powders that may disperse or to place the balance inside a fume hood if space permits. We recommend all synthetic chemistry procedures be conducted in a fume hood to limit airborne exposure. Furthermore, iron(III)-acetylacetonate falls under GHS classification H332 for acute toxicity; students should be supervised closely while handling this powder to ensure they follow proper safety protocols and avoid unnecessary exposure.

Day 1 of this laboratory protocol utilizes vacuum and Schlenk lines to carry out an oxygen-free reaction. The students should utilize vacuum grease when assembling the glassware to ensure proper evacuation and avoid introduction of oxygen during the reaction. The laboratory assistant should ensure that the Schlenk line is properly connected and the air-line is at an acceptable pressure before a student or student team begins their reaction. Nitrogen is sufficient for the air-line.

Throughout the laboratory, this procedure utilizes multiple flammable solvents, such as reagent alcohol, hexane, ethanol, and tetrahydrofuran. Care should be taken when handling these chemicals. The instructor and laboratory staff must ensure that storage protocols and liquid and solid waste streams that can accommodate these solvents are in place.

Day 1: Magnetic Nanoparticle Synthesis

Day 1 Overview

On Day 1 of this lab, students will synthesize iron oxide nanoparticles and isolate them via centrifugation (Figure S3). Students will:

- Set up the air-sensitive reaction to synthesize the particles
- Heat the reaction vessel to induce particle reflux
- Clean the particles with repeated centrifugation cycles


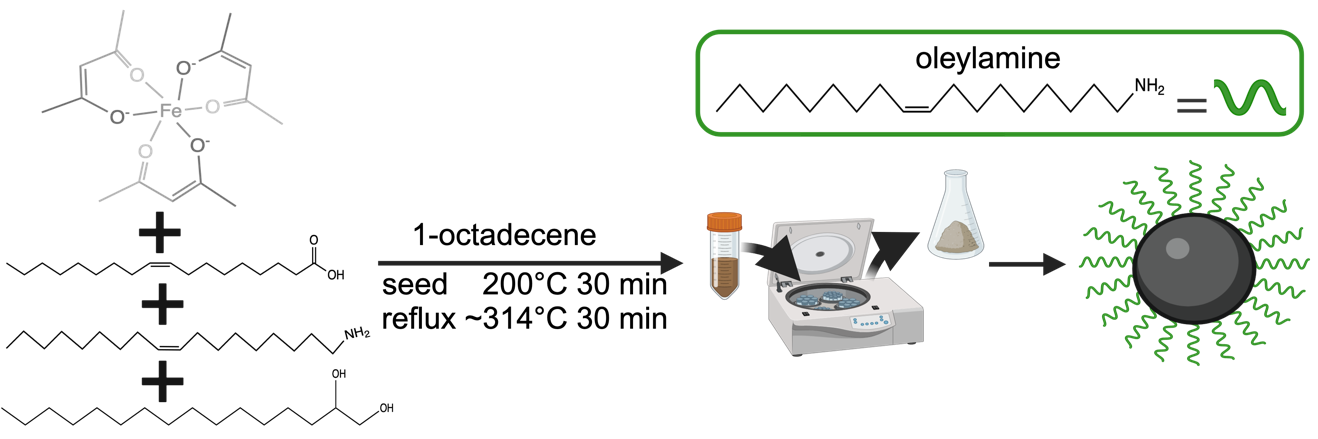


Figure S3. Overview of magnetic nanoparticle synthesis and cleaning. This figure was prepared using BioRender.

Day 1 Equipment, Materials, and Chemicals

The equipment (Table S5), materials (Table S6), and chemicals (Table S7) required for the Day 1 protocol are shown below. Note that the listed sizes for glassware and measurement tools as well as the quantities of chemicals assume the 80 mL batch size indicated in the Student Manual.

| Table S5. Equipment Required for the Day 1 Protocol | |
| --- | --- |
| Equipment | Number Needed |
| Fume hood | 1 |
| Electronic balance with 0.01 g resolution | 1 |
| Schlenk line | 1 |
| Stir plate | 1 |
| Heating mantle | 1 |
| Inconel or stainless steel thermocouple | 1 |
| Thermocouple adapter | 1 |
| Temperature controller | 1 |
| Centrifuge | 1 |
| Vacuum desiccator (optional) | 1 |
| Ring stand | 1 |

| Table S6. Materials and Consumables Required for the Day 1 Protocol | |
| --- | --- |
| Materials and Consumables | Number Needed |
| 250 mL three-neck round-bottom flask | 1 |
| 250 mL beaker | 1 |
| Egg-shaped magnetic stir bar | 1 |
| Rubber septa | 2 |
| 5 mL syringe | 2 |
| Hypodermic needle | 1 |
| Condenser, pump, container, and tubing | 1 |
| Luer-lock needle | 1 |
| Glassware clips | 3 |
| Metal hose clamps | 1 |
| 100 mL graduated cylinder | 1 |
| Vacuum grease | ~1 mL |
| 50 mL centrifuge tube | 3 |
| Neodymium block magnet | 1 |

| Table S7. Chemicals Required for the Day 1 Protocol assuming a Batch Size of 80 mL of 1-octadecene | | | |
| --- | --- | --- | --- |
| State | Chemical | Role | Required Mass/Volume^a^ |
| Solid | Fe(acac)_3_ | Reactant | 2.82 g |
| Solid | 1-2 hexadecanediol | Reactant | 5.17 g |
| Liquid | 1-octadecene | Reaction solvent | 80 mL |
| Liquid | Oleic acid | Reactant | 7.57 mL |
| Liquid | Oleylamine | Reactant | 7.90 mL |
| Liquid | Reagent alcohol | Quenching the reaction | 160 mL |
| Liquid | Hexane | Resuspending particles during centrifugation^b^ | ~100 mL per centrifugation cycle |
| Liquid | Reagent alcohol | Filling centrifuge tubes^b^ | ~50 mL per centrifugation cycle |
| ^a^Students should have calculated these values as part of their prelab preparations.  ^b^The required volumes of these chemicals will be dependent on the number of centrifugation cycles required to clean the particles. It is best to have extra volume of these chemicals easily on hand and available for use. | | | |

Day 1 Synthesis Protocol

The primary reagents used were obtained from TCI chemicals (oleylamine. oleic acid, 1,2-hexadecanediol, Fe(acac)_3_, Mn(acac)_3_, and Co(acac)_3_), Sigma-Aldrich (1-octadecene and tetrahydrofuran), and BeanTown Chemical (iodomethane). Solvents were obtained from VWR (hexane, ethanol, and reagent alcohol). All reagents were used as-received.

To synthesize the Fe_3_O_4_ particles, measure and add all solid and air-inert liquid reagents (1-octadecene, 1,2-hexadecanediol, and Fe(acac)_3_) and the stir bar into the three-neck round-bottom flask.

**Possible Variation:** To synthesize doped particles, Co(acac)_3_ and Mn(acac)_3_ can be added in a 1:2 ratio with the Fe(acac)_3_ respectively. For shorter lab periods, the laboratory assistant can measure out and combine the reagents in advance.

Then fill the pump container with water and connect the pump to the condenser so that water flows through the condenser jacket. At this point, the pump should not be turned on. Set up the heating mantle on the stir plate (Figure S4). Use vacuum grease and glassware clips to secure the round-bottom flask to the bottom of the condenser and the valve (in closed position) to the top of the condenser (Figure S4). The round-bottom flask should be nested in the heating mantle. Connect the valve to nitrogen flow from the Schlenk line and seal off the remaining two necks with rubber septa.

Note that students may not have had previous exposure to air-free methods in their chemistry curriculum and may not know how to operate a Schlenk line. The laboratory assistant needs to carefully check that vacuum grease and glassware clips are used at every joint and have been applied properly. It is easy for students to miss one or multiple of these. Once the laboratory assistant has confirmed the glassware is properly set up, the students should gather for a demonstration of the purge-pulse procedure. A helpful reference for proper Schlenk Line set-up and use can be found in this article by Borys.^10^


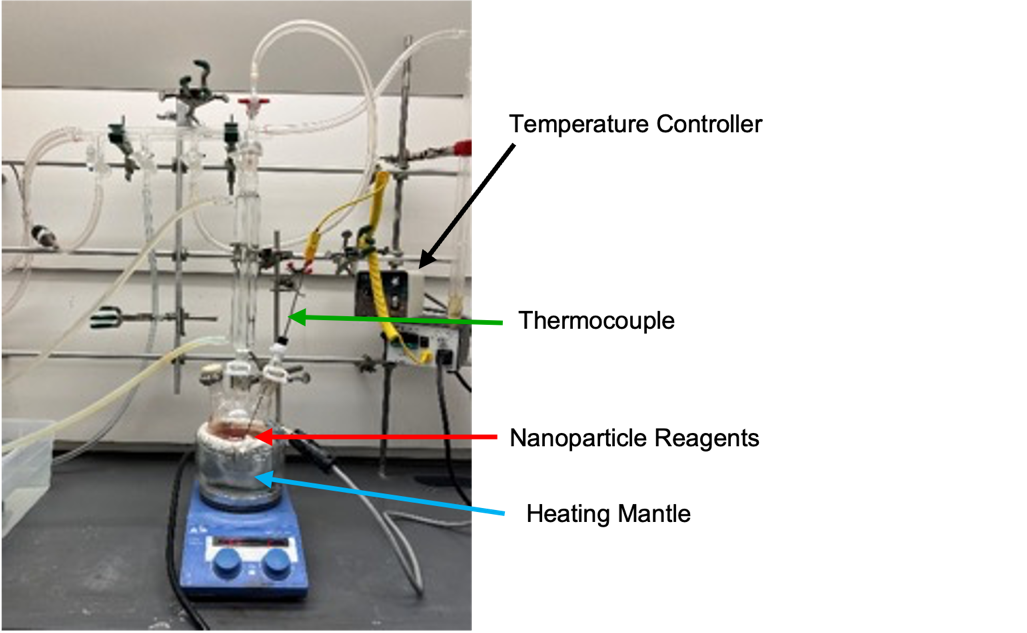


Figure S4. Three-neck round bottom flask placed on top of heating mantle with a condenser attached to the middle neck and rubber septa sealing the outer necks. The thermocouple with the appropriate adapter and the heating mantle are connected to temperature controller.

To promote safe laboratory operating conditions, the laboratory assistant should show students proper use of a Schlenk line by running a demonstration purge-pulse cycle. Students will use the knobs of the valve above the condenser tube and the Schlenk line to perform three pulse-purge cycles to cleanse the atmosphere in the system.

First, nitrogen will be added into the system through the condenser (pulse step) (Figure S5). This process is followed by purging with the vacuum line (purge step). Student will repeat the pulse-purge cycle two more times, and finish with the valve above the condenser in its closed position.


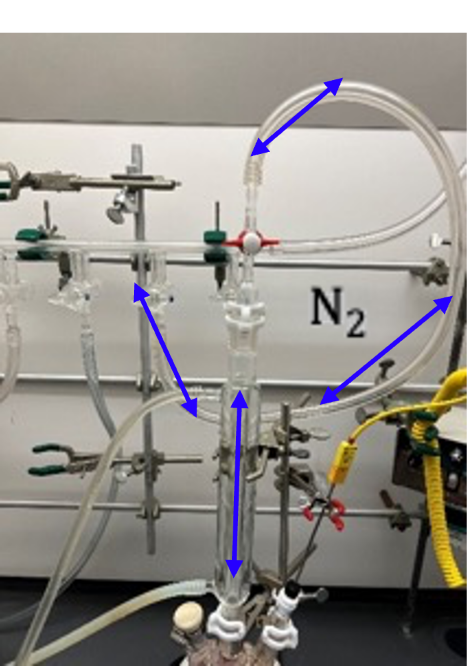


Figure S5. Overview of chemistry setup with reaction glassware inside a fume hood. Note the valves on the Schlenk line, the vertical condenser valve in the closed position, and that vacuum grease has been applied inside all tapered joints as well as safety clips where necessary.

From a different outlet on the Schlenk line, students will flow nitrogen through a needle into the left septum and use a hypodermic needle on the right septum to vent. To remove residual oxygen, students will bubble nitrogen through the reaction mixture for 20 minutes. Once the reaction vessel is devoid of oxygen, the air-sensitive reagents (oleylamine and oleic acid) can be introduced into the system via syringe.

At this point, it is important that the laboratory assistant walk around the room to confirm that all students have the Schlenk line valve set to the nitrogen position, that septa with metal hose clamp are properly inserted, and that all joints have vacuum grease and glassware clips applied before students begin heating the reaction.

After the laboratory assistant checks the reaction setup, students will begin heating until the reaction temperature reaches 200 ºC. In our experiments, the reaction reaches 200 ºC after approximately 30 minutes. Reaching this temperature marks the start of the “seed phase,” and the temperature should be controlled at this value for 30 minutes to allow particle nucleation.

After the isothermal 30-minute seed phase has elapsed, continue heating the vessel until the reaction begins to reflux; clouds of vapor will begin shooting up the condenser (Figure S6a). In our experiences, this occurred around 314 °C. Once refluxing begins, students should wrap aluminum foil around the reaction vessel to minimize heat loss to the environment while wearing heat-resistant gloves (Figure S6b). The laboratory assistant should supervise this process as the glassware poses a significant hazard for burns due to the high temperature of the reaction.

Allow the reaction to reflux for 30 minutes to facilitate particle growth. This second heating stage at the solvent reflux temperature serves to grow the nanoparticle nucleation sites into particles of the desired size. After 30 minutes have elapsed, the laboratory assistant should help students remove the heating mantle using heat-resistant gloves and allow the reaction to cool. Again, the glassware will be very hot at this point and there is a risk of students being burned.


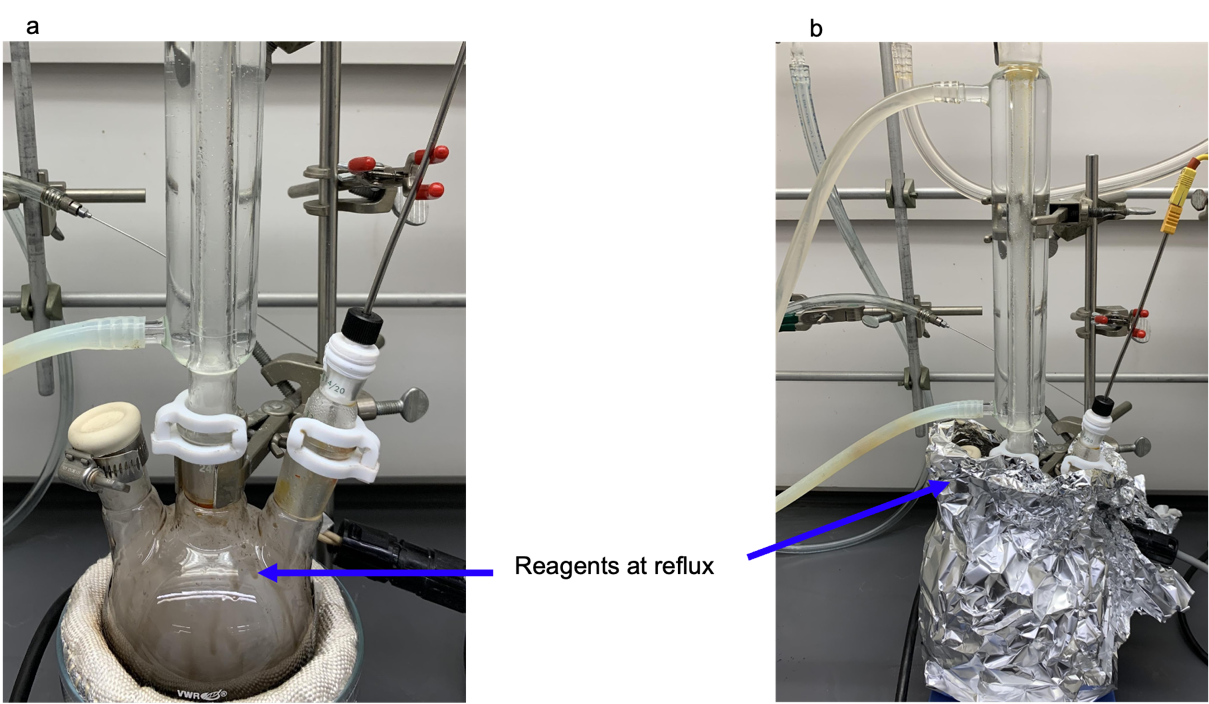


Figure S6. (a) Reaction in full reflux with cloud of vapor inside the condenser. (b) The round bottom flask covered with aluminum foil to minimize heat loss to environment during reflux.

The reaction should cool to 40 ºC within 30 minutes, after which students will quench the reaction by adding reagent alcohol at a ratio of double the original batch size; for the 80 mL batch size indicated in the Student Manual and demonstrated in Figures S4-S6, 160 mL of reagent alcohol is added. The contents of the flask will turn a hazy brown color after addition of the reagent alcohol, indicating a polarity change that lowers the solubility of the particles. The reaction will not proceed at this point.

**Possible Variation:** Based on our past results, the quenched dispersion can be stored in the presence of air for at least one week. This serves as a convenient stopping point if time is running short during a lab period. In this case, to remain on schedule with the rest of the lab, it would be best for the laboratory assistant to finish the protocol, including centrifuging the particles to clean and isolate them and subsequently drying the particles.

To clean the particles, fill enough centrifuge tubes to hold all the quenched product (Figure S7a). Centrifuge for 15 minutes at room temperature at 6000 RPM. After centrifuging is complete, remove the supernatant. A magnet may be held to the base of the tube to ensure only the supernatant is discarded. After discarding the supernatant, re-suspend the pellet of particles inside the centrifuge tube using the minimum volume of hexane possible. Then, combine the hexane/particle mixture in a beaker and note the volume. Add reagent alcohol to this beaker until the mixture turns cloudy, generally adding 50% of the starting volume, to increase the polarity of the mixture and encourage precipitation of the particles. Separate the hexane/particle/reagent alcohol mixture into counter-balanced tubes and repeat the 15-minute, room-temperature, 6000-RPM centrifuge cycle. After the first cycle, the supernatant should have a dark orange color (Figure S7b).

Students should complete as many centrifuge cycles as time permits. If needed, the laboratory assistant should conduct the remainder of the cleaning to achieve a nearly clear supernatant as shown in Figure S7c. The particles are deemed clean when the supernatant remaining after a centrifuge cycle has a very slight yellow tint and the particles have settled along the tube walls (Figure S7c).

Lastly, the particles should be dried to form a powder (Figure S7d). The samples can be left to dry over a week inside the fume hood in a covered centrifuge tube, but if faster (~2 hour) drying is desired, a vacuum desiccator can be used.


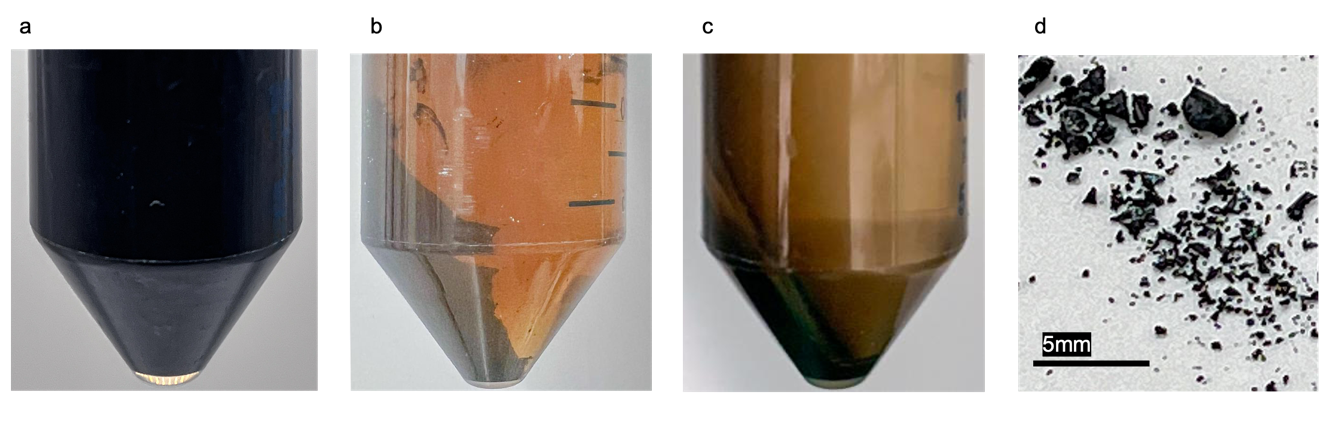


Figure S7. Series of images of particles at different stages in the process: (a) immediately after synthesis before centrifugation, (b) immediately after one round of centrifugation, (c) after cleaning when clear supernatant is obtained, and (d) after drying using a vacuum desiccator.

Day 2: Ligand Stripping and Sample Preparation

Day 2 Overview

In Day 2 of this lab, students will strip the surface of the particles, perform a ligand exchange, and prepare samples of their particles for characterization in Day 3 (Figure S8). As noted in the article text, there are several approaches for sample characterization. While this manual details characterization using SEM, DLS, and magnetophotometry, the sample preparation methods performed on Day 2 produce samples that are compatible with all characterization techniques mentioned (dried and suspended particles).

Students will:

- Prepare a sample for dynamic light scattering (DLS) by creating a dilute dispersion of nanoparticles in hexane
- Prepare a sample for scanning electron microscopy (SEM) by drop-casting nanoparticles onto a silicon wafer chip
- Modify the surface of the particles by stripping their ligands to prepare them for magnetic characterization


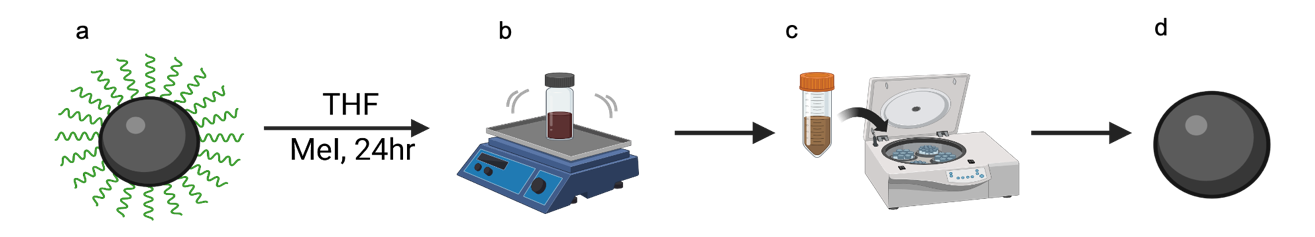


Figure S8. Overview of the iron oxide nanoparticle ligand stripping procedure. Begin by taking the (a) dry particles from Day 1 and (b) adding tetrahydrofuran (THF) and iodomethane (MeI), and then stirring overnight. (c) Transfer the stripped particles into a centrifuge tube and centrifuge. Decant the supernatant and allow the particle pellet formed at the bottom of the centrifuge tube to dry leaving (d) stripped iron oxide nanoparticles. This figure was prepared using BioRender.

Day 2 Equipment, Materials, and Chemicals

The equipment (Table S8), materials (Table S9), and chemicals (Table S10) required for the Day 2 protocol are shown below. Note that the listed sizes for glassware and measurement tools as well as the quantities of chemicals assume the 80 mL batch size indicated in the Student Manual.

| Table S8. Equipment Required for the Day 2 Protocol | |
| --- | --- |
| Equipment | Number Needed |
| Shake / inversion table | 1 |
| Fume hood | 1 |
| Vacuum desiccator | 1 |
| Sonicator | 1 |
| 100-1000 µL pipette | 1 |
| 250 mL beaker | 1 |

| Table S9. Materials and Consumables Required for the Day 2 Protocol | |
| --- | --- |
| Materials and Consumables | Number Needed |
| 10 mL glass vial | 2 |
| Disposable cuvette with lid | 1 |
| Si wafer chip (1 cm x 1 cm) | 1 |
| 50 mL glass vial | 1 |
| 50 mL centrifuge tube | 1 |
| Disposable tips for 100-1000 µL pipette | ≥5 |

| Table S10. Chemicals Required for the Day 2 Protocol | | | |
| --- | --- | --- | --- |
| State | Chemical | Role | Required Mass/Volume |
| Solid | Fe_3_O_4_ nanoparticles | DLS sample | 0.4 mg |
| Liquid | Hexane (HPLC grade) | DLS sample solvent | 4 mL |
| Solid | Fe_3_O_4_ nanoparticles | SEM sample | 5 mg |
| Liquid | Ethanol | SEM sample drop-cast solvent | 2 mL |
| Solid | Fe_3_O_4_ nanoparticles | Nanoparticles to be stripped | 50 mg |
| Liquid | Tetrahydrofuran (THF) | Ligand exchange reagent | 20 mL |
| Liquid | Iodomethane (MeI) | Ligand exchange reagent | 0.7 mL |

Day 2: Ligand Stripping and Sample Preparation Protocol

The dried and suspended nanoparticle samples must be prepared for analysis. Completing this sample preparation is the focus of Day 2.

Both SEM-EDX and DLS analysis use the dry powdered sample from Day 1 (Figure S8a) with no modification. When preparing samples for DLS analysis, students will prepare a 2 mL dispersion using a solvent that is compatible with the available cuvettes. The dispersions should contain iron oxide particles at a concentration no higher than 0.1 mg/mL.

In the Student Manual, students are instructed to dissolve the nanoparticles in HPLC-grade hexane to create 4 mL of a 0.1 mg/mL dispersion and use disposable PMMA cuvettes; this is how we obtained the DLS data shown in Figure 3 in the main text. We found that mixing the dispersion with a vortex mixer followed by sonication resulted in the best suspension of the nanoparticles into the solvent. Following this protocol, students should prepare the dispersion in a small glass vial and then pipette 2 mL of that dispersion into a cuvette for DLS analysis.

To prepare the SEM sample, students will dissolve iron oxide nanoparticles in ethanol at a concentration of 2.5 mg/mL and then drop-cast the dispersion onto clean silicon chips with a 1-micron thermal oxide layer. Two drops of the dispersion should be sufficient to coat a 1 cm x 1 cm square silicon chip. Again, we recommend using sonication to fully dissolve the particles during the suspension process. Note that ethanol is preferred over hexane (as used in the DLS sample) to avoid additional carbon deposition from the hexane. We have observed similar results when using silicon chips from a wafer with a 2-micron thermal oxide layer or a native oxide layer instead of a 1-micron thermal oxide layer.

**Possible Variation:** As electron microscopes can greatly vary in their operating voltage and resolution, the sample preparation may need to be modified for the specific microscope available. We recommend that the instructor or laboratory assistant test and optimize these imaging parameters as needed prior to the students completing the laboratory.

To conduct magnetic response testing, the oleylamine ligands on the surface of the particles need to be removed. Students will put 50 mg of the dried sample in a glass vial, add 20 mL of tetrahydrofuran (THF) and 0.7 mL of iodomethane (MeI), and dissolve the nanoparticles via sonication. The laboratory assistant should ensure that the students measure THF and iodomethane inside a fume hood as these reagents pose an inhalation hazard. To mix the dispersion without introducing a stir bar, utilize a shake table, inversion table, or any other non-contact mixing equipment (Figure S8b). Let the particle/THF/MeI dispersion mix overnight. This is the last step the students will complete as part of Day 2.

The rest of the ligand exchange protocol should be completed by the laboratory assistant. Once mixing is complete, the laboratory assistant will add an equivalent volume of reagent alcohol to the mixture and centrifuge for 15 minutes at 6000 RPM to collect the particles at the bottom of the tube (Figure S8c). The laboratory assistant will then remove the supernatant and dry the particles once more. The samples can be left to dry over a week inside the fume hood in a covered centrifuge tube, but if faster (~2 hour) drying is desired, a vacuum desiccator can be used. After this surface treatment, the dry particles will be referred to as stripped particles (Figure S8d). These stripped particles will be used to prepare the magnetophotometry (MAP) sample for magnetic characterization in Day 3.^11^

Note that the nanoparticle dispersions can be stored for a month in a closed glass vial sealed with parafilm and the SEM samples can be stored for a month if placed inside of a desiccator with active desiccant.

Day 3: Nanoparticle Characterization and Data Analysis

Day 3 Overview

In Day 3 of this lab, students will prepare samples and characterize them using an SEM and a MAP (Figure S9). The laboratory assistant should characterize samples using DLS prior to this session and provide students with data. Students will:

- Finish sample dispersion preparation for magnetic characterization
- Quantify particle size using Dynamic Light Scattering (DLS)
- Analyze particle size and shape using Scanning Electron Microscopy (SEM)
- Estimate particle magnetic susceptibility using magnetophotometry (MAP)


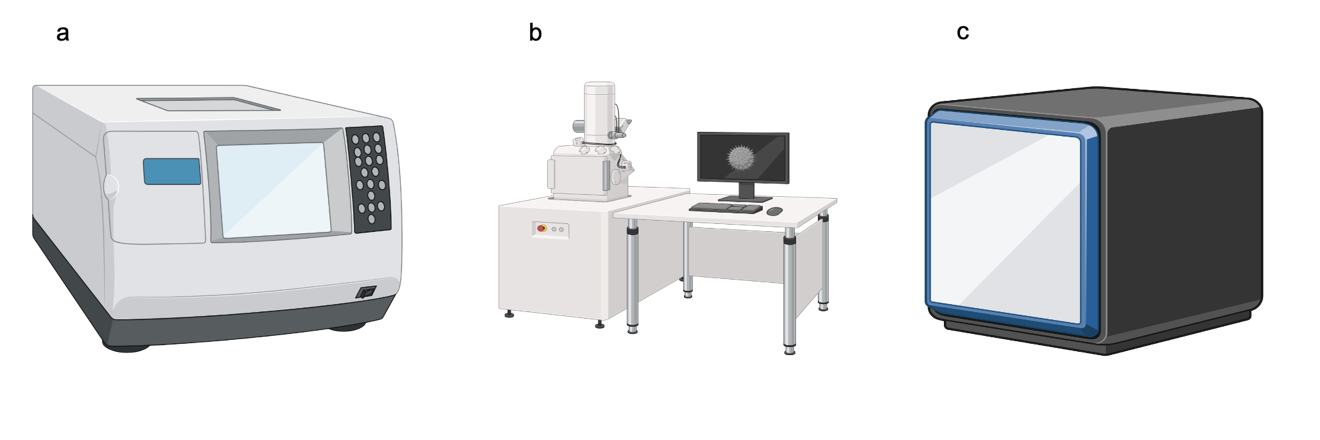


Figure S9. Characterization techniques used in this laboratory include (a) Dynamic Light Scattering (DLS), (b) Scanning Electron Microscopy (SEM), and (c) magnetophotometry (MAP). This figure was prepared using BioRender.

Day 3 Equipment, Materials, and Chemicals

The equipment (Table S11), materials (Table S12), and chemicals (Table S13) required for the Day 3 protocol are shown below. Note that the listed sizes for glassware and measurement tools as well as the quantities of chemicals assume the 80 mL batch size indicated in the Student Manual.

| Table S11. Equipment Required for the Day 3 Protocol | |
| --- | --- |
| Equipment | Number Needed |
| Vortex | 1 |
| Sonicator | 1 |
| 100-1000 µL pipette | 1 |

| Table S12. Materials and Consumables Required for the Day 3 Protocol | |
| --- | --- |
| Materials and Consumables | Number Needed |
| 10 mL glass vial | 1 |
| Disposable cuvette with lid | 1 |
| Disposable tips for 100-1000 µL pipette | ≥2 |

| Table S13. Chemicals Required for the Day 3 Protocol | | | |
| --- | --- | --- | --- |
| State | Chemical | Role | Required Mass/Volume |
| Solid | Fe_3_O_4_ stripped nanoparticles | MAP sample | 0.4 mg |
| Liquid | Ethanol | MAP sample solvent | 4 mL |

Day 3 Characterization Protocol

Due to equipment access constraints, we had a laboratory assistant perform DLS measurements on student samples and provide the data to the students. It is up to the course instructor to decide whether to provide the students with a plot of the DLS results or provide them with the acquisition results and have them analyze the data. To perform DLS measurements, we utilized a Wyatt Technology Mobius system equipped with a 532 nm laser and performed 20 acquisitions to calculate particle size (Figure S9a). We repeated the DLS measurements 3 times to confirm particle size. In our tests, a concentration of 0.1 mg/mL provided the optimal combination of signal and accuracy without particles settling and affecting the measurement. Note that it is expected that particles will settle to the bottom of the vial after a few minutes; we found that 2-3 inversions were sufficient to re-disperse the particles for long enough to run the 20-acquisition measurement. Capping the sample is recommended to prevent hexane from evaporating during the measurement, as this can damage the instrument and lead to erroneous results.

Under the guidance of the laboratory assistant, students will conduct SEM measurements of particle size using the sample prepared on Day 2 and interpret the results. For the SEM data shown in Figure 3 of the main text, a Hitachi TM3000 with an accelerating voltage of 5 kW and NanoSEM 450 at an accelerating voltage of 15 kV were used (Figure S9b). Samples prepared for SEM can be dried using an active vacuum for 1 hour to eliminate ethanol residue. The laboratory assistant should supervise students at all times while they utilize the SEM to ensure correct operation.

For magnetic analysis, as mentioned, we used an open-source MAP in our work. This simple and inexpensive system allows for a quantitative assessment of the nanoparticle magnetic response, improving the quality of the analysis.^11^ To prepare a sample for magnetic analysis via the MAP, students should create a 4 mL dispersion at a concentration of 0.1 mg/mL of ethanol, using sonication to dissolve the particles (Figure S9c). It is important that the particles fully dissolve and disperse through the solvent, turning the dispersion an opaque brown. We found experimentally that this concentration yielded the best compromise in detecting particle magnetic response and maintaining acceptable light transmission at the start of MAP testing.

**Possible Variation:** Should some of these methods of characterization not be available, a list of alternative techniques is listed in Table S14 in the Possible Variations section.

Possible Variations

Nanoparticle Synthesis

**Nanoparticle Doping**

The focus of the protocol is to synthesize iron oxide nanoparticles. As indicated in Section 2, this protocol can be easily adjusted to synthesize Co- and Mn-doped particles by adding Co(acac)_3_ or Mn(acac)_3_ in a 1:2 ratio with the Fe(acac)_3_ respectively.

**Seed Time**

While we outlined the procedure for nanoparticle synthesis with a seed time of 30 minutes, we have successfully made nanoparticles with seed times up to 2.5 hours. Longer seed times result in larger particles. However, the size distribution is less controlled.

**Reaction Solvent**

The above procedure utilizes 1-octadecene as the reaction solvent. In theory, any non-reactive solvent with a boiling point higher than 200°C would be compatible. We have successfully recreated this reaction using phenyl ether as the reaction solvent which results in smaller particles.

In this procedure, we added reagent alcohol to the reaction mixture to promote gravitational separation by changing the polarity of the mixture. After exploring acetone, methanol, and ethanol (instead of reagent alcohol) with the 1-octadecene system, we found reagent alcohol had the best separation performance. If the reaction solvent is replaced, one will need to explore different separation solvents. For example, we found that a 50:50 mixture of ethanol and acetone is satisfactory for separation within the phenyl ether reaction system.

Characterization

A list of characterization methods for analyzing relevant properties is provided in Table S14, including the methods used in this protocol. A potential sample type is also included in this table.

| Table S14. Parameters of Interest and Possible Characterization Methods | | | |
| --- | --- | --- | --- |
| Parameter Measured | Measurement Method | Data visualization | Type of Sample |
| Particle size | Dynamic Light Scattering (DLS) | Histogram | Coated, suspended |
| Particle size | Transmission Electron Microscope (TEM) | Image | Coated, dried |
| Particle size | Scanning Electron Microscope (SEM) | Image | Coated, dried |
| Particle size | Atomic Force Microscopy (AFM) | 2.5D image | Coated, dried |
| Dopant concentration | Energy Dispersive X-ray Spectroscopy (EDX/EDS) | Spectra | Coated, dried |
| Dopant concentration | X-Ray Diffractometry (XRD) | Spectra | Coated, dried |
| Magnetic response | Magnetic Resonance Imaging (MRI) | Image |  |
| Magnetic response | Magnetophotometry (MAP) | Graph | Stripped, suspended |
| Magnetic response | SQUID | Graph | Coated, dried |

Data Analysis

An emerging area of engineering is computer-aided image analysis using free programs like Python or R in combination with ImageJ. One extension to the procedure is to automate the identification and quantification of the particle size and morphology by developing and implementing an edge-detection approach. This type of program could also be written in MATLAB. We did not pursue this method during our testing, but it could be included in a modification of this laboratory procedure and provide a straightforward strategy to incorporate computer science principles into chemical engineering, materials science, and chemistry coursework.

Educational Assessment

Student Volunteer Background

The student volunteers were all undergraduate students in the Viterbi School of Engineering at the University of Southern California. Three students were first-year undergraduates, four were second-year undergraduates, and one was a third-year undergraduate. Given this academic year range, the students had diverse transcripts. Table S15 summarizes several courses that might be considered pre-requisites and the percentage of this student set that had completed the courses at USC or received transfer credit. These results are self-reported.

| Table S15. Potential Coursework that could be Considered as a Pre-Requisite | | |
| --- | --- | --- |
| Course | Relevance to Present Lab | % of students |
| General Chemistry and laboratory (entire year) | Basics of reactions, lab skills, chemical equations and rates | 100% |
| Organic Chemistry and laboratory (1 semester) | Advanced lab skills and synthetic reactions, material purification | 50% |
| Physical Chemical Measurement Laboratory | Advanced measurement methods and data analysis | 13% |
| Analytical Chemistry | Advanced data analysis, sample purification | 0% |
| Physics I and laboratory | Understanding signal processing | 100% |
| Physics II and laboratory | Understanding magnetism | 63% |
| Physics III and laboratory | Understanding light scattering (SEM, DLS, and MAP operation) | 0% |

As can be seen in Table S15, while all students had completed General Chemistry and Physics I, the completion rate for higher level courses quickly dropped off. This minimum level of background training is notable, given the subsequent assessment findings.

Assessment Strategy

To assess the educational effectiveness of this laboratory, several approaches were used. Students completed pre-laboratory (written) questions. Summative and formative assessments were also performed during and immediately after the completion of the laboratory. Finally, a reflective assessment was performed six months after the laboratory. The results from each of these assessment methods are presented and discussed.

Pre-laboratory Assessment

The student prelab questions were written to ensure that the students understand what they would be doing in lab that day prior to attending the lab session. The questions and answers are included in the accompanying Student Assessment and Solutions documents. The questions are primarily formatted as short answer questions to promote critical thinking about the concepts presented. The exception is the Day 1 pre-laboratory, which includes several basic calculations related to the synthesis to simulate the types of calculations a student would be expected to do in a research setting. Some questions pose hypothetical situations that students may not have enough experience to correctly solve, but these are primarily intended to prepare the students for the lab and promote students thinking about the lab instead of simply executing out the steps.

Formative Assessment

Formative assessment was performed through active discussions during the instructional sessions. Taking a Socratic approach, the laboratory assistant guided the small student group discussions before, during, and after the laboratory to ensure that the key concepts and learning goals for that day were achieved. Example questions used on the different days include:

**Day 1: Magnetic Nanoparticle Synthesis**

- Why does nitrogen need to be bubbled through the solvent before starting the reaction?
- Before beginning the reaction, we conduct a series of pulse and purge steps using the Schlenk line. Why is this important?
- What role does the condenser serve in managing the reaction?

**Day 2: Ligand Stripping and Sample Preparation**

- What is the role of the iodomethane in the ligand exchange process?
- Why are we not using the Schlenk line while performing the ligand exchange process?
- Why do you think we are using stripped nanoparticles when preparing samples for SEM? What would happen if we did not strip these ligands?

**Day 3: Nanoparticle Characterization and Data Analysis**

- We utilize both DLS and SEM to perform size analysis; why do you think we utilize both?
- Why do you think we utilize different solvents when preparing samples for DLS/SEM/MAP analysis?
- We notice some batch-to-batch variation in the size and magnetic susceptibility of the nanoparticles produced by the different groups. What is one processing step that could influence these measurements?

Summative Assessment

At the end of the three-day series, we asked a series of closed-form and open-form questions. The results are below.

The closed form question was very simple: “Please indicate your agreement with the following series of statements.” The results are shown in Figure S10. As can be seen, the students universally responded that the goals and objectives were clear, they learned something new, and they enjoyed the lab. However, they also felt that the rigor of the lab could be increased. To aid in addressing this weakness, we have included several possible variations that will allow future implementations of this lab to be uniquely adopted for specific student groups.


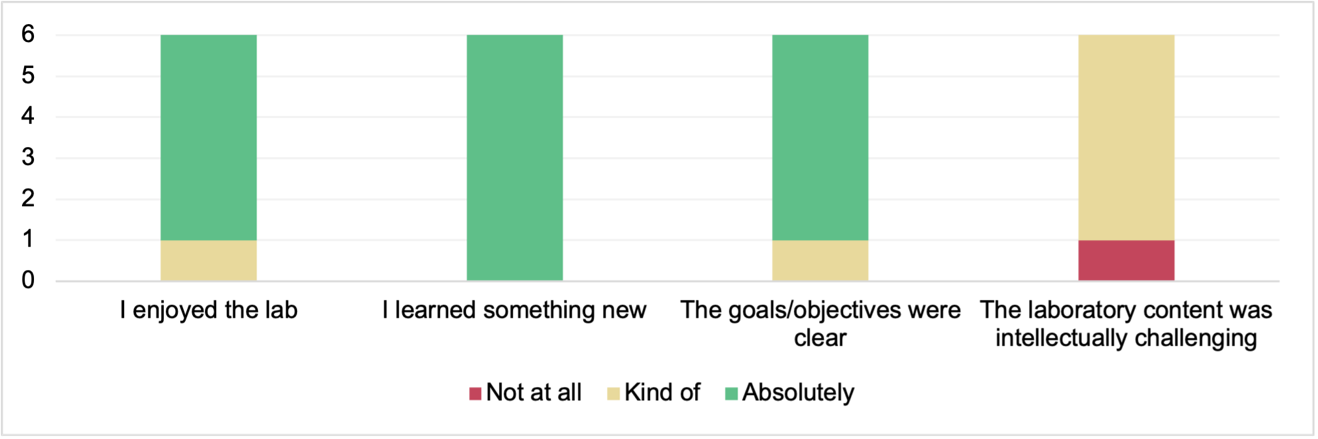


Figure S10. Student post-laboratory poll showing the results of the six participants who completed the survey out of the 9-student cohort.

The majority of the assessment was dedicated to the four open-form questions detailed below. The responses are summarized immediately following each question along with one student’s response.

**1. What motivated you to complete this experiment?**

The majority of students indicated that their primary motivator was the opportunity to learn about nanoparticle synthesis and characterization. Nearly all students indicated an interest in careers in the nanotechnology area, and they were excited to have the opportunity to get hands-on learning in a relevant area. One student explicitly mentioned that they were losing interest in their course laboratories, and this laboratory sounded exciting.

“One of the main reasons I applied to USC under the chemical engineering department is because I am hugely interested in participating in the nanotechnology emphasis. Thus, I believed this lab would tell me more about the industry as well as what it's like to analyze nanoparticles. I wanted to see first-hand how these nanoparticles are made and analyzed, as well as learn more about the uses of these nanoparticles. After this experiment, I can confidently say I am much more interested in taking part in the nanotechnology industry.”

**2. What worked best? What did you enjoy the most?**

A universal theme in all comments was an appreciation for a sense of teamwork that the lab required. Additionally, there were several positive comments about the detailed nature of the lab manual and the opportunity to learn new pieces of equipment that have applications in many fields.

“I liked the undergraduate students that I was working with and the PhD students that guided us through the process. I also learned a lot of different procedures in the lab and learned new concepts about the techniques used to study our particles (SEM, Magnetophotometer, Condensers) and it was cool seeing the nanoparticles we made.”

**3. What didn’t work? What aspects should be improved?**

The primary concern was related to the timing of the three sessions. Specifically, the students suggested trying to re-organize the experiment such that the laboratory assistant does more of the work so that there is less down-time (or waiting time) for the students.

“I think some parts of the lab could have been more consolidated. I felt like there were times where we were sitting and waiting when perhaps some other part of the lab could have been done.”

**4. What is your overall assessment? Should this lab (or a similar lab) be integrated into an upper division undergraduate class?**

Universally, the students enjoyed the experiments and measurements, and the students were strongly supportive of this lab being integrated into their coursework and undergraduate education. One student suggested that the lab could be modified or adapted for a first-year course as well. Following on this point, there was some concern about what course it would best fit, given the interdisciplinary nature of the content.

However, all students responded positively about their experience and the knowledge learned.

“The lab should be integrated into an upper division undergraduate class because it combines so much different content neatly into one experiment, and will definitely enhance student understanding.”

Reflective Assessment

Six months after completing the laboratory series, we asked a series of closed-form and open-form questions to assess knowledge gain and retention as well as perceived laboratory impact. Notably, all students completed the reflective survey, indicating a high level of engagement and interest in this effort. The results are summarized below (Figures S11-S14), including one unedited response for each of the open form questions.

As can be seen in the responses to the following four closed-form questions, upon reflection, the procedures have had the largest impact on the students’ understanding and proficiency with characterization instrumentation and with air-sensitive chemical synthesis apparatus.


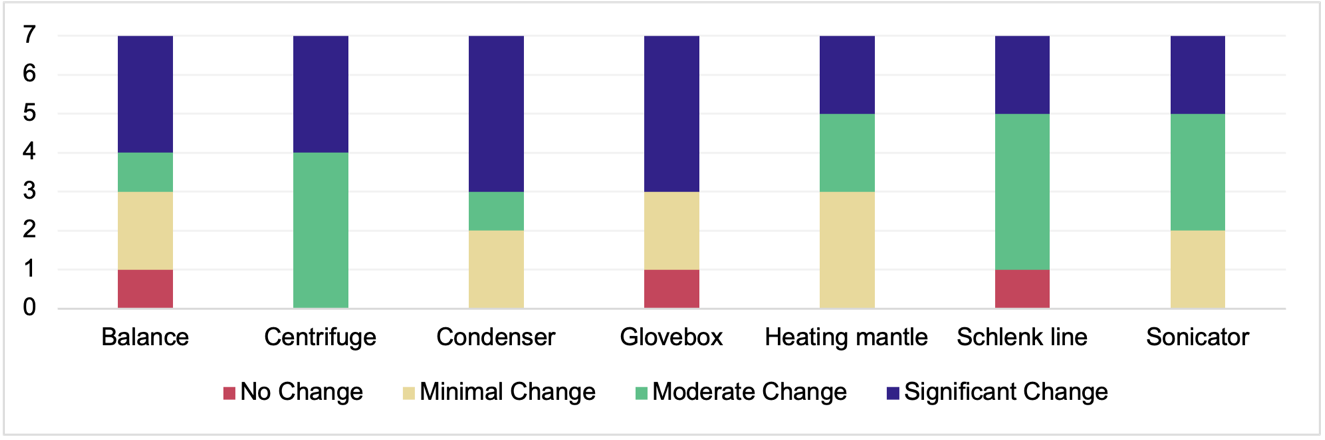


Figure S11. Student responses to “How did the material and procedures performed in the lab increase your proficiency with (or ability to perform) the following Air-Sensitive Chemistry Procedures?”


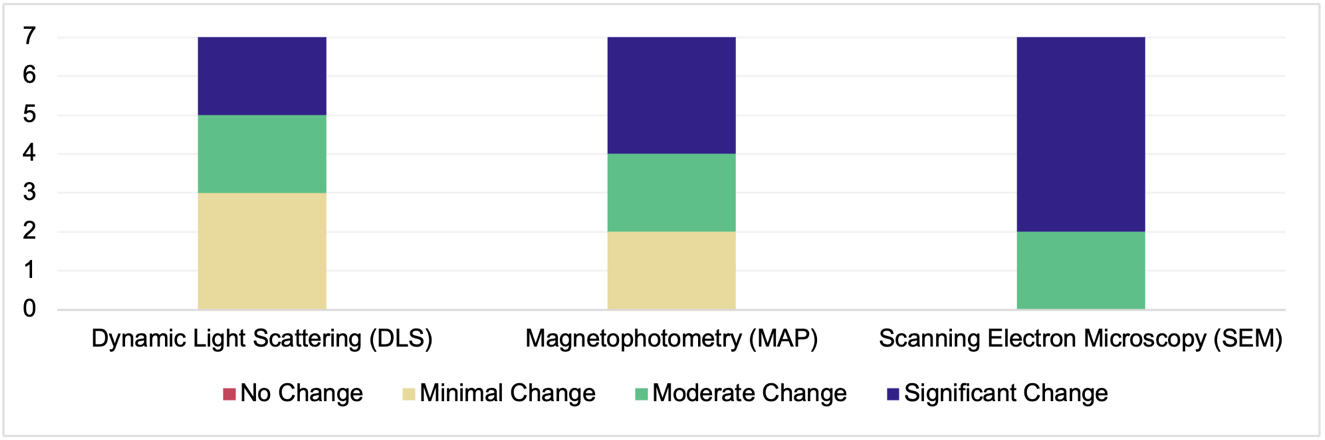


Figure S12. Student responses to “How did the information discussed in the lab increase your technical understanding of the following Materials Characterization Methods?”


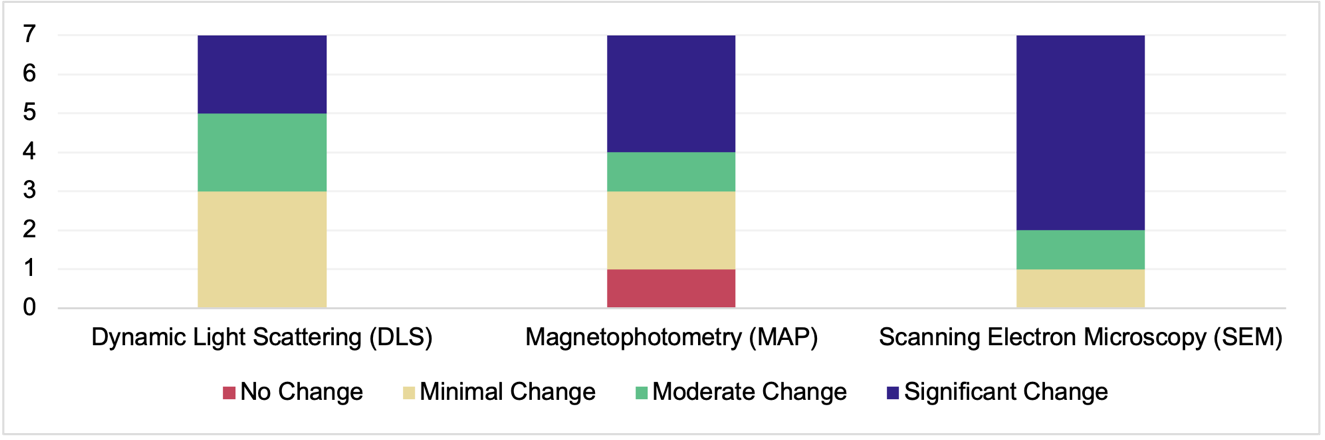


Figure S13. Student responses to “How did the materials and procedures performed in the lab increase your proficiency with (or ability to perform) the following Materials Characterization Methods?”


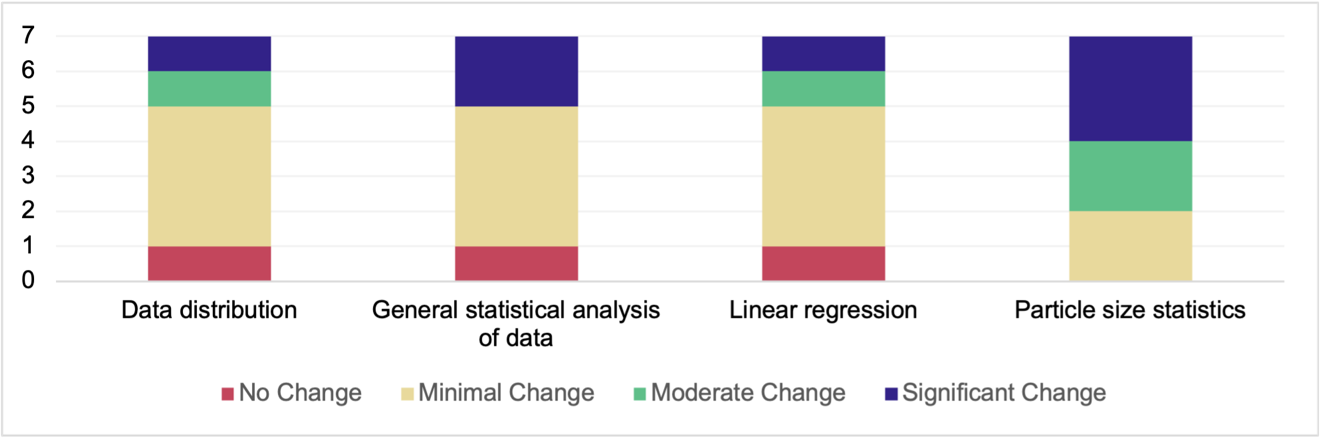


Figure S14. Student responses to “How did the information discussed in the lab increase your technical understanding of the following Data Analysis Methods?”

The pair of open-form questions were designed to encourage students to reflect on the concepts that they learned during the laboratory and to evaluate how they anticipate applying the skills and knowledge in subsequent experiences.

1. **How did this lab contribute to an increase in your understanding of these concepts or other concepts?**

A pair of themes emerged in the feedback that were not captured in the closed-form questions. First, the students realized the importance of safety and performing measurements in a safe manner. Second, several students commented that many of the methods introduced were completely unfamiliar to them, to the extent that they did not know that the instrument existed before performing the lab. An example comment from a student is below:

“Before this lab, I had a general idea that some of these concepts existed, like centrifugation and magnetic response testing. However, I had never really understood what these concepts are or why they are important, and this lab helped enhance my understanding in these concepts. This lab taught me the importance of controlled temperature and quenching when making new particles. Before this lab, I did not even know what quenching was. Moreover, the use of SEM, DLS, magnetic testing, etc. helped me analyze particles in a very detailed way that I did not know was previously possible, where we could analyze data mathematically and visually.”

1. **How do you envision the skills that you learned in this lab being applied to your future coursework or in other research/career settings?**

The answers to this question were the most diverse and insightful of the assessment. In only 6 months, the student cohort has already applied the knowledge and skills in their current courses, research positions, and/or internships. Additionally, as indicated in the representative comment below, the students have found value in a diverse range of fields well-outside of the standard materials-curriculum.

“The lab helped me develop my problem solving skills especially since it was in a subject that I was less familiar with, i.e. chemistry. In my future, I don't know how much the chemistry specific skills I learned will be used much due to my personal career path, but my increased technical understanding and increased ability to perform the specified data analysis methods will without a doubt be useful. For example, I am taking a machine learning course this semester, and all of those skills probably will be used to complete assignments and projects. Also, as a potential software engineer in industry, regardless of what type of software or company I work for, I am sure it will be helpful to have proficient skills in those data analysis methods.”

References

(1) Wu, L.; Mendoza-Garcia, A.; Li, Q.; Sun, S. Organic Phase Syntheses of Magnetic Nanoparticles and Their Applications. *Chem. Rev.* **2016**, *116* (18), 10473–10512. https://doi.org/10.1021/acs.chemrev.5b00687.

(2) Delgado, T.; Villard, M. Spin Crossover Nanoparticles. *J. Chem. Educ.* **2022**, *99* (2), 1026–1035. https://doi.org/10.1021/acs.jchemed.1c00990.

(3) Kim, D. K.; Zhang, Y.; Voit, W.; Rao, K. V.; Muhammed, M. Synthesis and Characterization of Surfactant-Coated Superparamagnetic Monodispersed Iron Oxide Nanoparticles. *Journal of Magnetism and Magnetic Materials* **2001**, *225* (1), 30–36. https://doi.org/10.1016/S0304-8853(00)01224-5.

(4) Chi, Y.; Yuan, Q.; Li, Y.; Tu, J.; Zhao, L.; Li, N.; Li, X. Synthesis of Fe3O4@SiO2–Ag Magnetic Nanocomposite Based on Small-Sized and Highly Dispersed Silver Nanoparticles for Catalytic Reduction of 4-Nitrophenol. *Journal of Colloid and Interface Science* **2012**, *383* (1), 96–102. https://doi.org/10.1016/j.jcis.2012.06.027.

(5) Tanaka, S.; Kaneti, Y. V.; Septiani, N. L. W.; Dou, S. X.; Bando, Y.; Hossain, Md. S. A.; Kim, J.; Yamauchi, Y. A Review on Iron Oxide-Based Nanoarchitectures for Biomedical, Energy Storage, and Environmental Applications. *Small Methods* **2019**, *3* (5), 1800512. https://doi.org/10.1002/smtd.201800512.

(6) Fruntke, A.; Behnke, M.; Stafast, L. M.; Träder, T.; Dietel, E.; Vollrath, A.; Weber, C.; Schubert, U. S.; Wilke, T. Targeted Drug Delivery: Synthesis of Smart Nanocarriers for School Chemistry Education. *J. Chem. Educ.* **2023**, *100* (2), 751–759. https://doi.org/10.1021/acs.jchemed.2c00422.

(7) Rattanakit, P. Open Inquiry-Based Laboratory Project on Plant-Mediated Green Synthesis of Metal Nanoparticles and Their Potential Applications. *J. Chem. Educ.* **2021**, *98* (12), 3984–3991. https://doi.org/10.1021/acs.jchemed.1c00300.

(8) Reddy, L. H.; Arias, J. L.; Nicolas, J.; Couvreur, P. Magnetic Nanoparticles: Design and Characterization, Toxicity and Biocompatibility, Pharmaceutical and Biomedical Applications. *Chem. Rev.* **2012**, *112* (11), 5818–5878. https://doi.org/10.1021/cr300068p.

(9) Lee, N.; Yoo, D.; Ling, D.; Cho, M. H.; Hyeon, T.; Cheon, J. Iron Oxide Based Nanoparticles for Multimodal Imaging and Magnetoresponsive Therapy. *Chem. Rev.* **2015**, *115* (19), 10637–10689. https://doi.org/10.1021/acs.chemrev.5b00112.

(10) Borys, A. M. An Illustrated Guide to Schlenk Line Techniques. *Organometallics* **2023**, *42* (3), 182–196. https://doi.org/10.1021/acs.organomet.2c00535.

(11) Scholtz, A.; Paulson, J.; Nunez, V.; Armani, A. M. Open-Source Magnetophotometer (MAP) for Nanoparticle Characterization. **2023**. https://doi.org/10.48550/arXiv.2401.01903.
