# Supplementary material for: Synthesis and Characterization of Superparamagnetic Iron Oxide Nanoparticles: A Series of Laboratory Experiments
Source: J Chem Educ. 2024 Apr 9;101(5):2039–44. doi: 10.1021/acs.jchemed.3c00996 (PMC11097384; doi:10.1021/acs.jchemed.3c00996)
Supplement: Supplementary file 3 — ed3c00996_si_003.pdf [file ed3c00996_si_003.pdf]

Supporting Information for:

Synthesis and Characterization of Superparamagnetic Iron Oxide Nanoparticles: A Series of Laboratory Experiments

Armando D. Urbina[1+], Hari Sridhara[1+], Alexis Scholtz[2+], Andrea M. Armani[1,2,3] *

[1] Mork Family Department of Chemical Engineering and Materials Science, University of Southern California, Los Angeles, CA 90089, USA

[2] Alfred E. Mann Department of Biomedical Engineering, University of Southern California, Los Angeles, CA 90089, USA

[3] Ellison Institute of Technology, Los Angeles, CA 90064, USA

[+] These authors contributed equally.

*aarmani@eit.org

Synthesis and Characterization of Superparamagnetic Iron Oxide Nanoparticles

Students' Manual

TABLE OF CONTENTS

Background *3*

Overview of experiment *5*

Hazards *6*

Day 1: Magnetic Nanoparticle Synthesis *7*

Day 1 Overview 7

Day 1 Equipment, Materials, and Chemicals 8

Day 1 Synthetic protocol 10

Day 2: Ligand Stripping and Sample Preparation *15*

Day 2 Overview 15

Day 2 Equipment, Materials, and Chemicals 16

Day 2 Ligand Stripping and Sample Preparation Protocol 18

Day 3: Nanoparticle Characterization and Data Analysis *20*

Day 3 Overview 20

Day 3 Equipment, Materials, and Chemicals 21

Day 3 Characterization Protocol 22

References *24*

BACKGROUND

Nanomaterials have recently helped advance electronics, medicine, and manufacturing technologies. Thus, it is important for chemical engineering and materials science curricula to prepare students for these careers through exposure to nanomaterials and training on relevant skills and techniques. Iron oxide nanoparticles are chosen for this teaching exercise both because of the novelty of this material in educational settings and their exceptional magnetic response, low biotoxicity, and ease of fabrication[1–4] that make them so useful. Further, iron oxide nanoparticles have vast applications in technologies such as imaging contrast agents, catalysis, and data storage.[5–9]

These applications leverage the specific magnetic properties of iron oxide nanoparticles, which exhibit paramagnetism. Unlike many magnetic materials which rely on collective, long-range magnetic order to achieve an intrinsic magnetic response, paramagnetic and diamagnetic materials are considered nonmagnetic in the absence of an external magnetic field. However, in the presence of a field, their magnetic domains align in a single direction, as seen in Figure S1, and the particle exhibits a magnetic response. The electron pairing determines if a material is diamagnetic or paramagnetic, which governs the sign of the magnetic susceptibility. Symmetric or spherical iron oxide nanoparticles demonstrate their paramagnetic response due to their unpaired electrons and have a positive magnetic susceptibility. If the particles are small enough, they may exhibit superparamagnetism, which is when particles exhibit very short magnetic relaxation response times, resulting in superior paramagnetic properties.

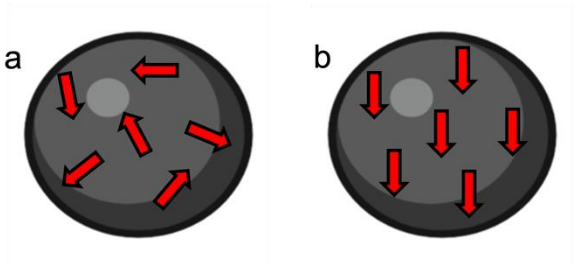

Figure S1. Paramagnetic nanoparticle (a) before and (b) during exposure to an external magnetic field pointing towards the bottom of the page.

Given the diversity of applications for paramagnetic iron oxide nanoparticles, it is useful to measure their size, composition, and magnetic response.

As part of this series of experiments, you will develop the following skills:

- Air-sensitive chemistry (ex. Schlenk line, glove box)
- Laboratory techniques (ex. centrifuge, sonicator)
- Synthesis equipment proficiency (ex. heating mantle, condenser)
- Characterization techniques (ex. DLS, SEM, magnetophotometry)
- Data analysis methods (ex. linear regression, distribution, size statistics)

OVERVIEW OF EXPERIMENT

The purpose of this series of laboratories is to demonstrate how the superparamagnetic behavior of iron oxide nanoparticles depends on the synthesis conditions and material doping. Day 1 focuses on the synthesis (Figure S2a-c), Day 2 on the surface treatment and sample preparation of the nanoparticles (Figure S2d-f), and Day 3 on the nanoparticle characterization and analysis (Figure S2g-i).

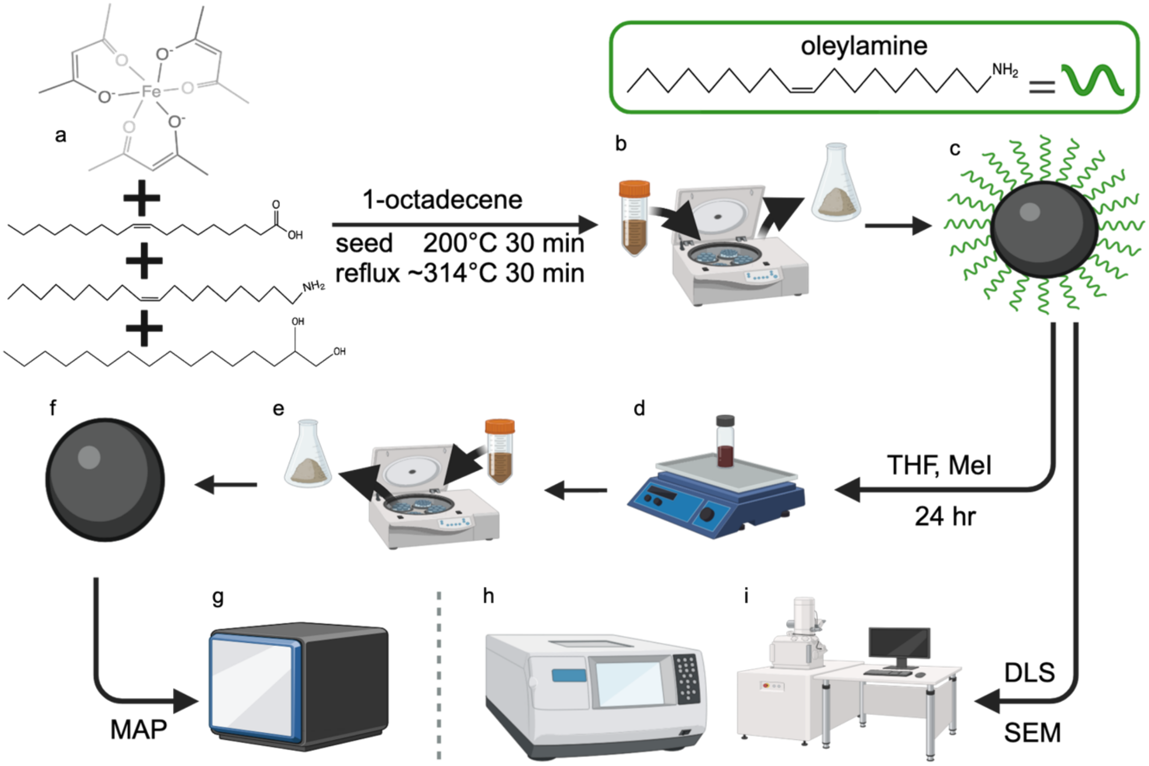

Figure S2. Overview of the full laboratory procedure. (a-c) Day 1 consists of nanoparticle synthesis including (a) the Fe_3O_4 synthesis reaction, (b) isolating the iron oxide nanoparticles by centrifuge, and (c) drying the iron oxide nanoparticles coated with oleylamine ligands. (d-f) Day 2 includes (d) the stripping of nanoparticles by iodomethane (MeI), (e) centrifugation to isolate the stripped iron oxide nanoparticles and (f) drying the stripped Fe_3O_4 nanoparticles. (g-i) Characterization techniques used in Day 3 include (g) magnetophotometry (MAP) using stripped nanoparticles, (h) Scanning Electron Microscopy (SEM) using oleylamine-coated nanoparticles, and (i) Dynamic Light Scattering (DLS) using oleylamine-coated nanoparticles. This figure was prepared using BioRender.

HAZARDS

A concrete understanding of laboratory safety is essential preparation for careers in science and engineering. This laboratory procedure involves use of numerous hazardous chemicals and requires the use of appropriate mitigation measures. You should wear proper personal protective equipment (PPE), including a high-temperature lab coat, goggles, and nitrile or heat-resistant (when necessary) gloves, in accordance with standard chemistry practices. Beyond standard safety practices and initial general laboratory safety training, this laboratory also involves more extensive work with powder, vacuum and Schlenk lines, and flammable solvents, all of which is detailed below. A trained laboratory assistant should be present throughout the procedure to supervise all steps and assist when necessary.

When working with powders, it is important for you to measure your powder reagents in a well-ventilated area to avoid accidental inhalation. It is preferable to have an exhaust snorkel above the electronic scale which will aspirate any powders that may disperse or to place the balance inside a fume hood if space permits. We recommend all synthetic chemistry procedures be conducted in a fume hood to limit airborne exposure. Furthermore, iron(III)-acetylacetonate falls under GHS classification H332 for acute toxicity; a laboratory assistant should supervise you closely while handling this powder to ensure you follow proper safety protocols and avoid unnecessary exposure.

Day 1 of this laboratory protocol utilizes vacuum and Schlenk lines to carry out an oxygen-free reaction. You should utilize vacuum grease when assembling the glassware to ensure proper evacuation and avoid introduction of oxygen during the reaction. The laboratory assistant should ensure that the Schlenk line is properly connected and the air-line is at an acceptable pressure before you begin your reaction. Nitrogen is sufficient for the air-line.

Throughout the laboratory, this procedure utilizes multiple flammable solvents, such as reagent alcohol, hexane, ethanol, and tetrahydrofuran. Take care when handling these chemicals and be sure to dispose of these solvents in the appropriate liquid and solid waste streams.

DAY 1: MAGNETIC NANOPARTICLE SYNTHESIS

Day 1 Overview

On Day 1 of this lab, you will synthesize iron oxide nanoparticles and isolate them via centrifugation (Figure S3). You will:

- Set up the air-sensitive reaction to synthesize the particles
- Heat the reaction vessel to induce particle reflux
- Clean the particles with repeated centrifugation cycles

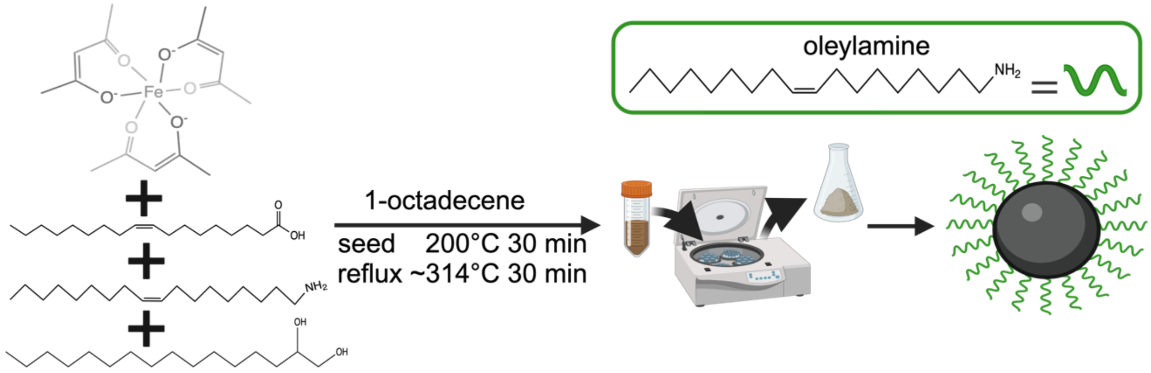

Figure S3. Overview of magnetic nanoparticle synthesis and cleaning. This figure was prepared using BioRender.

Day 1 Equipment, Materials, and Chemicals

You will need the following pieces of equipment (Table S1), materials and consumables (Table S2), and chemicals (Table S3) for the Day 1 protocol. Use Table S3 to record the quantities of chemicals you measure and use during Day 1.

Table S1. Equipment Required for the Day 1 Protocol

Equipment	Number Needed
Fume hood	1
Electronic balance with 0.01 g resolution	1
Schlenk line	1
Stir plate	1
Heating mantle	1
Inconel or stainless steel thermocouple	1
Thermocouple adapter	1
Temperature controller	1
Centrifuge	1
Vacuum desiccator (optional)	1
Ring stand	1

Table S2. Materials and Consumables required for the Day 1 Protocol

Materials and Consumables	Number Needed
250 mL three-neck round-bottom flask	1
250 mL beaker	1
Egg-shaped magnetic stir bar	1
Rubber septa	2
5 mL syringe	2
Hypodermic needle	1
Condenser, pump, container, and tubing	1
Luer-lock needle	1
Glassware clips	2
Metal hose clamps	2
100 mL graduated cylinder	1
Vacuum grease	~1 mL
50 mL centrifuge tube	3
Neodymium block magnet	1

Table S3. Chemicals Required for the Day 1 Protocol.

State	Chemical	Role	Required Mass/Volume[a]	Measured Mass/Volume[b]
Solid	$Fe(acac)_3$	Reactant		
Solid	1-2 hexadecanediol	Reactant		
Liquid	1-octadecene	Reactant		
Liquid	Oleylamine	Reactant		
Liquid	Oleic acid	Reactant		
Liquid	Reagent alcohol	Quenching the reaction	160 mL	n/a
Liquid	Hexane	Resuspending particles during centrifugation[c]	~100 mL per centrifugation cycle	n/a
Liquid	Reagent alcohol	Filling centrifuge tubes[c]	~100 mL per centrifugation cycle	n/a

[a]For all reactants, be sure to include the masses and volumes of each reagent that you calculated in the prelab.

[b]Use the final column of this table to record the measured masses and volumes of each reagent that you use for the reaction.

[c]The required volumes of these chemicals will be dependent on the number of centrifugation cycles required to clean the particles.

Day 1 Synthetic protocol

1.1. Measure and add all solid reagents ($Fe(acac)_3$ and 1,2-hexadecanediol), the air-inert liquid reagent (1-octadecene), and the stir bar into the three-neck round-bottom flask.

1.1.a. Be sure to record the measured amount of each chemical on Table S3.

1.2. Fill the pump container with water and connect the pump to the condenser so that water flows through the condenser jacket. At this point, the pump should not be turned on.

1.3. Set up the heating mantle on the hot plate.

1.4. Use vacuum grease and glassware clips to secure the middle septa of the round-bottom flask to the bottom of the condenser and the valve (in closed position) to the top of the condenser (Figure S4). The bottom of the round-bottom flask should be nested in the heating mantle.

1.5. Connect the valve to nitrogen flow from the Schlenk line and seal off the remaining two necks with rubber septa (Figure S4).[10]

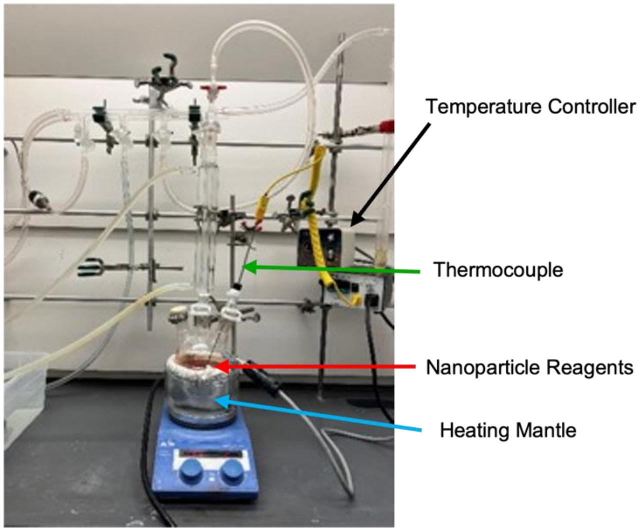

Figure S4. Overview of chemistry setup inside a fume hood. Three-neck round bottom flask placed on top of heating mantle with a condenser attached to the middle neck and rubber septa sealing the outer necks. The thermocouple, pierced through one of the rubber septa, and the heating mantle are connected to temperature controller.

1.6. Once the three necks on the round bottom flask are connected, open the nitrogen flow from the Schlenk line to the condenser.

1.7. Use the knobs of the valve above the condenser tube and the Schlenk line to perform a series of pulse-purge cycles to cleanse the atmosphere in the system (Figure S5).

1.7.a. First, perform the pulse step by opening the nitrogen valve connected to the condenser for one minute. The vacuum line should be closed.

1.7.b. After one minute, perform the purge step by closing the nitrogen line and opening the vacuum line for one minute.

1.7.c. Repeat the pulse-purge cycle two more times.

1.7.d. Finish by closing the vacuum line and opening the nitrogen line.

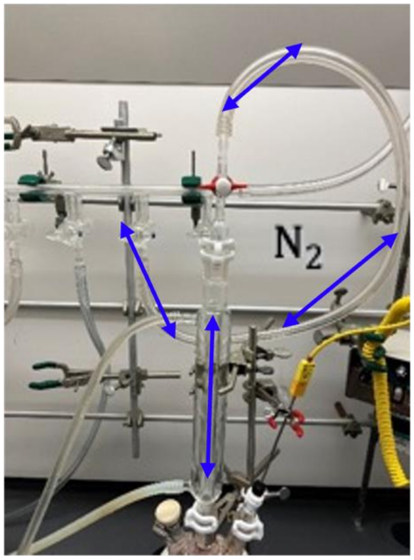

Figure S5. Overview of chemistry setup with reaction glassware inside a fume hood. Note the valves on the Schlenk line, the vertical condenser valve in the closed position, and that vacuum grease has been applied inside all tapered joints as well as safety clips where necessary.

1.8. From a different outlet on the Schlenk line, flow nitrogen through a needle and into the left septum. Use a hypodermic needle on the right septum to vent.

1.9. To remove residual oxygen, bubble nitrogen through the reaction mixture for 20 minutes.

1.10. Insert the temperature probe (thermocouple) into the right septa (Figure S6a).

1.11. Once the reaction vessel is devoid of oxygen, add the air-sensitive reagents (oleylamine and oleic acid) into the system via syringe.

1.12. Turn condenser pump on.

1.13. Check with your laboratory assistant that your reaction setup is properly assembled before moving to the next step.

1.14. Connect the heating mantle and temperature probe to the temperature controller (Figure S4). Begin heating the reaction vessel until the internal temperature (as measured by the probe) reaches 200°C (about 30 minutes).

1.15. Once the temperature reaches 200°C, keep the temperature constant for 30 minutes to begin the "seed phase," when the particles nucleate.

1.16. After 30 minutes, increase the temperature until the reaction begins to reflux and you see clouds of vapor begin to shoot up the condenser (Figure S6a). This may occur above 300°C.

1.17. Once refluxing begins, wrap aluminum foil around the reaction vessel to minimize heat loss to the environment while wearing heat-resistant gloves (Figure S6b). Keep the temperature constant for 30 minutes to facilitate particle growth.

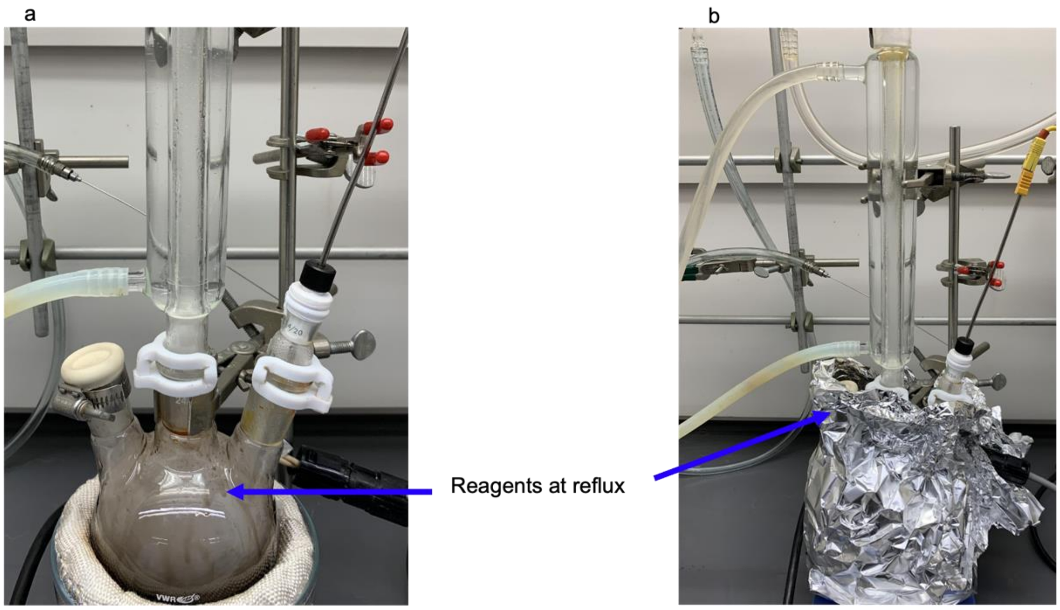

Figure S6. (a) Reaction in full reflux with cloud of vapor inside the condenser. (b) Round bottom flask covered with aluminum foil to minimize heat loss to environment.

1.18. After refluxing for 30 minutes, remove the flask from the heating mantle and allow it to cool. The glassware will be very hot so be sure to wear heat-resistant gloves and take care when handling the reaction flask.

1.19. Once the internal temperature of the reaction drops below 40°C, add 160 mL of reagent alcohol (double the initial batch size) to quench the reaction. The reagent alcohol will increase the polarity of the dispersion and promote separation of the nanoparticles from unreacted reagents. The contents of the reaction flask should turn a hazy brown color. No further reaction should occur past this point.

1.20. To clean the particles, fill enough centrifuge tubes to hold all the quenched product (Figure S7a).

1.21. Create counterweight tubes filled with water to match the weight of each tube of product to balance the centrifuge. The weights should match within ~5%. Be sure to insert the matched pairs of product-counterweight in opposite spots within the centrifuge to avoid damaging the instrumentation.

1.22. Centrifuge the particles for 15 minutes at 6000 RPM at room temperature.

1.23. After centrifuging, remove the supernatant via pipette. A magnet may be held to the base of the tube to ensure only the supernatant is discarded. After this first cycle, the supernatant should have a dark brown color (Figure S7b).

1.24. To re-suspend the particles, add as little hexane as possible to dissolve the pellet of particles accumulated at the bottom of the centrifuge tube. Transfer the hexane and particle mixture to a beaker and note the volume.

1.25. Add a reagent alcohol to the beaker containing the hexane/particle mixture until the mixture turns cloudy. The volume added should be around 50% of the noted hexane/particle volume. The reagent alcohol will increase the polarity of the mixture and encourage precipitation of the particles.

1.26. Repeat the 15-minute, room temperature, 6000-RPM centrifuge cycle as many times as lab time permits or until the particles are clear. The particles are deemed clean when the supernatant remaining after a centrifuge cycle has a very slight yellow tint and the particles have settled along the tube walls (Figure S7c).

1.27. After the last centrifugation cycle, do not re-suspend the particles.

1.28. To dry the particles and form a powder, insert the centrifuge tube into a vacuum desiccator and leave under vacuum until dry (Figure S7d). The drying step will take at least 2 hours.

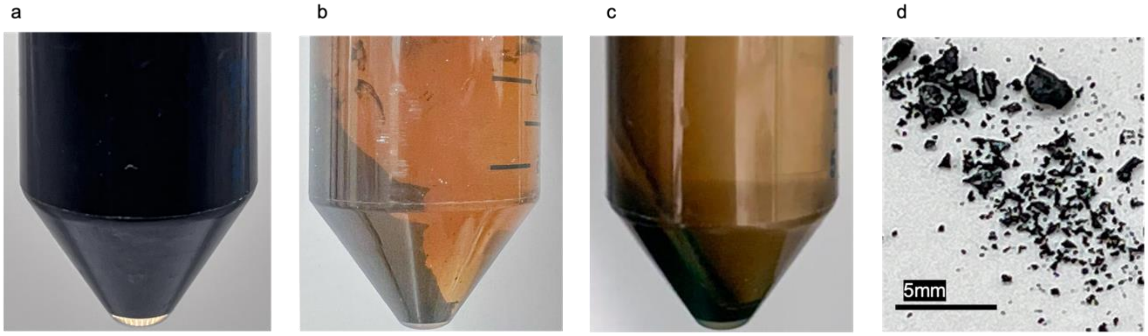

Figure S7. Series of images of particles at different stages in the process: (a) immediately after synthesis before centrifugation, (b) immediately after one round of centrifugation, (c) after cleaning, when clear supernatant is obtained, and (d) after drying using a vacuum desiccator.

DAY 2: LIGAND STRIPPING AND SAMPLE PREPARATION

Day 2 Overview

On Day 2 of this lab, you will strip the surface of the particles, perform a ligand exchange, and prepare samples of your particles for characterization on Day 3 (Figure S8). You will:

- Prepare a sample for dynamic light scattering (DLS) by creating a dilute dispersion of nanoparticles in hexane
- Prepare a sample for scanning electron microscopy (SEM) by drop-casting nanoparticles onto a silicon wafer
- Perform a ligand exchange to strip particles to prepare them for magnetic characterization

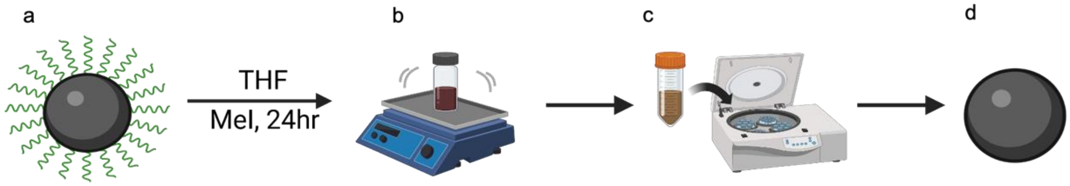

Figure S8. Overview of the iron oxide nanoparticle ligand stripping procedure. Begin by taking the (a) dry particles from Day 1 and (b) adding tetrahydrofuran (THF) and iodomethane (MeI), and then stirring overnight. (c) Transfer the stripped particles into a centrifuge tube and centrifuge. Decant the supernatant and allow the particle pellet formed at the bottom of the centrifuge tube to dry leaving (d) stripped iron oxide nanoparticles. This figure was prepared using Biorender.

Day 2 Equipment, Materials, and Chemicals

You will need the following pieces of equipment (Table S4), materials and consumables (Table S5), and chemicals (Table S6) for the Day 2 protocol. Use Table S6 to record the quantities of chemicals you measure and use during Day 2.

Table S4. Equipment Required for the Day 2 Protocol

Equipment	Number Needed
Shake / inversion table	1
Fume hood	1
Vacuum desiccator	1
Sonicator	1
100-1000 μL pipette	1
250 mL beaker	1

Table S5. Materials and Consumables Required for the Day 2 Protocol

Materials and Consumables	Number Needed
10 mL glass vial	2
Disposable cuvette with lid	1
Si wafer chip (1 cm x 1 cm)	1
50 mL glass vial	1
50 mL centrifuge tube	1
Disposable tips for 100-1000 μL pipette	≥5

Table S6. Chemicals Required for the Day 2 Protocol

State	Chemical	Role	Required Mass/Volume	Measured Mass/Volume[a]
Solid	Fe_3O_4 nanoparticles	DLS sample	0.4 mg	
Liquid	Hexane (HPLC grade)	DLS sample solvent	4 mL	
Solid	Fe_3O_4 nanoparticles	SEM sample	5 mg	
Liquid	Ethanol	SEM sample drop-cast solvent	2 mL	
Solid	Fe_3O_4 nanoparticles	Nanoparticles to be stripped	50 mg	
Liquid	Tetrahydrofuran (THF)	Ligand exchange reagent	20 mL	
Liquid	Iodomethane (MeI)	Ligand exchange reagent	0.7 mL	

[a]Use the final column of this table to record the measured masses and volumes of each chemical that you use to prepare your samples.

Day 2 Ligand Stripping and Sample Preparation Protocol

First, prepare your DLS sample from the dry powdered sample with no modification. This requires 2 mL of dispersion at a concentration of ≤0.1 mg/mL. You will make 4 mL of a 0.1 mg/mL dispersion using hexane as a solvent.

2.1. Measure 0.4 mg of your dry powdered sample from Day 1 (Figure S7d) and add the nanoparticles to a 10-mL glass vial. Label the glass vial and record the amount measured in Table S6.

2.2. Measure 4 mL of HPLC-grade hexane and add to the glass vial containing your nanoparticles. Record the amount measured in Table S6.

2.3. Vortex and sonicate the glass vial until the nanoparticles are fully suspended and no longer visible within the dispersion. This may take ~3 minutes per vortex-sonicate cycle depending on the batch size and concentration of particles used. Repeat the vortex-sonicate cycle until the particles are fully suspended. In prior efforts this required between 5-10 cycles.

2.4. Pipette 2 mL of the dispersion into a plastic PMMA cuvette to be used for DLS.

While the DLS sample dispersion is sonicating, prepare your SEM sample. This requires depositing 1-2 drops of a nanoparticle dispersion onto silicon chips from an initial concentration of 2.5 mg/mL.

2.5. Measure 5 mg of your dry powdered sample from Day 1 and add the nanoparticles to a second 10 mL glass vial. Grinding down particle clumps into a fine dust at this point will make the particle suspension in step 2.7 faster. Label the glass vial and record the amount measured in Table S6.

2.6. Pipette 2 mL of ethanol and add to the glass vial containing your nanoparticles. Record the amount measured in Table S6.

2.7. Vortex and sonicate the glass vial until the nanoparticles are fully suspended and no longer visible within the dispersion. In previous experiments it has taken 10-15 minutes of sonication to completely dissolve particle clumps.

2.8. Obtain a clean silicon wafer from your graduate assistant.

2.9. Drop-cast the 2.5 mg/mL nanoparticle dispersion onto your clean silicon chip. Two drops should be sufficient to coat your wafer.

Finally, prepare your sample for magnetic testing. This requires removing the oleylamine ligands on the surface of the particles.

2.10. Measure 50 mg of your dry powdered sample from Day 1 and add the nanoparticles to a 50 mL glass vial. Label the glass vial and record the amount measured in Table S6.

2.11. Inside a fume hood, add 20 mL of tetrahydrofuran and 0.7 mL of iodomethane. Record the amount measured in Table S6.

2.12. Dissolve the nanoparticles by sonicating for 10 minutes.

2.13. Allow to the dispersion to mix overnight using the shake or inversion table.

At this point, the laboratory assistant will finish the remainder of your sample preparation by completing the following steps once the dispersion is done mixing:

- Centrifuge the dispersion for 15 minutes at room temperature at 6000 RPM
- Remove the supernatant
- Dry the particles

The resulting stripped particles will be ready for use in the magnetic characterization testing that you will complete on Day 3.

DAY 3: NANOPARTICLE CHARACTERIZATION AND DATA ANALYSIS

Day 3 Overview

On Day 3 of this lab, you will prepare samples and characterize them using DLS, SEM, and MAP (Figure S9). Your laboratory assistant has characterized your samples using DLS prior to this session and will provide the DLS data for you, but you will perform the SEM and MAP measurements. You will:

- Finish sample dispersion preparation for magnetic characterization
- Quantify particle size using Dynamic Light Scattering (DLS)
- Analyze particle size and shape using Scanning Electron Microscopy (SEM)
- Estimate particle magnetic susceptibility using magnetophotometry (MAP)[11]

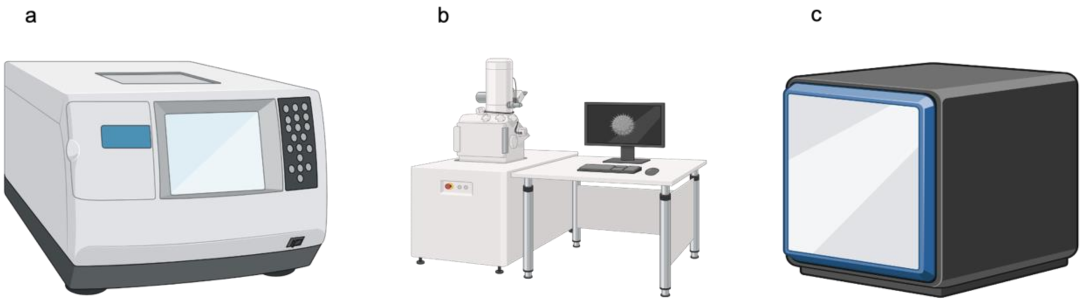

Figure S9. Characterization techniques used in this laboratory include (a) Dynamic Light Scattering (DLS), (b) Scanning Electron Microscopy (SEM), and (c) magnetophotometry (MAP). This figure was prepared using Biorender.

Day 3 Equipment, Materials, and Chemicals

You will need the following pieces of equipment (Table S7), materials and consumables (Table S8), and chemicals (Table S9) for the Day 3 protocol. Use Table S9 to record the quantities of chemicals you measure and use during Day 3.

Table S7. Equipment Required for the Day 3 Protocol

Equipment	Number Needed
Vortex	1
Sonicator	1
100-1000 µL pipette	1

Table S8. Materials and Consumables Required for the Day 3 Protocol

Materials and Consumables	Number Needed
10 mL glass vial	1
Disposable cuvette with lid	1
Disposable tips for 100-1000 µL pipette	≥2

Table S9. Chemicals Required for the Day 3 Protocol

State	Chemical	Role	Required Mass/Volume	Measured Mass/Volume[a]
Solid	Fe_3O_4 stripped nanoparticles	MAP sample	0.4 mg	
Liquid	Ethanol	MAP sample solvent	4 mL	

[a]Use the final column of this table to record the measured masses and volumes of each chemical that you use to prepare your samples.

Day 3 Characterization Protocol

3.1. Obtain your DLS data from your prepared Day 2 sample from your laboratory assistant. You will be responsible for analyzing this data in your lab report.

The dry stripped nanoparticles from Day 2 are used in the magnetic characterization measurements. Before performing the measurements, the samples must be prepared.

3.2. Measure 0.4 mg of your dry stripped nanoparticles from Day 2 and add the nanoparticles to a 10-mL glass vial. Label the glass vial and record the amount measured in Table S9.

3.3. Pipette 4 mL of ethanol and add to the glass vial containing your nanoparticles. Record the amount measured in Table S9.

3.4. Vortex and sonicate the glass vial until the nanoparticles are fully suspended and no longer visible within the dispersion (~10-15 minutes).

3.5. Pipette 2 mL of the nanoparticle dispersion into a PMMA cuvette.

Next you will carry out SEM and MAP characterization. Because the MAP measurements occur over time, you will run these tests simultaneously to using the SEM.

3.6. Set up the MAP test properties by entering a file name and a 25-minute test duration into the MAP instrument. Ensure that the magnet is pulled out from under the testing stage.

3.7. Shake or vortex the MAP sample for at least 30 seconds to ensure that the nanoparticles are evenly dispersed and then insert the cuvette into the MAP. Place the cover over the cuvette and start the measurement.

3.8. After 15 seconds, push the magnet into place below the cuvette.

3.9. Leave the MAP to continue running.

3.10. Repeat the test two more times for reproducibility. Between each test, vortex and sonicate the MAP sample for at least 30 seconds each to ensure that the nanoparticles are evenly dispersed.

This is important to ensure the particles do not retain magnetization, are not clumped, and are sufficiently redispersed.

3.11. While the MAP tests are running, your graduate assistant will train you on the SEM to obtain images of your prepared nanoparticle sample from Day 2.

REFERENCES

(1) Wu, L.; Mendoza-Garcia, A.; Li, Q.; Sun, S. Organic Phase Syntheses of Magnetic Nanoparticles and Their Applications. *Chem. Rev.* **2016**, *116* (18), 10473–10512. https://doi.org/10.1021/acs.chemrev.5b00687.

(2) Delgado, T.; Villard, M. Spin Crossover Nanoparticles. *J. Chem. Educ.* **2022**, *99* (2), 1026–1035. https://doi.org/10.1021/acs.jchemed.1c00990.

(3) Kim, D. K.; Zhang, Y.; Voit, W.; Rao, K. V.; Muhammed, M. Synthesis and Characterization of Surfactant-Coated Superparamagnetic Monodispersed Iron Oxide Nanoparticles. *Journal of Magnetism and Magnetic Materials* **2001**, *225* (1), 30–36. https://doi.org/10.1016/S0304-8853(00)01224-5.

(4) Chi, Y.; Yuan, Q.; Li, Y.; Tu, J.; Zhao, L.; Li, N.; Li, X. Synthesis of Fe3O4@SiO2–Ag Magnetic Nanocomposite Based on Small-Sized and Highly Dispersed Silver Nanoparticles for Catalytic Reduction of 4-Nitrophenol. *Journal of Colloid and Interface Science* **2012**, *383* (1), 96–102. https://doi.org/10.1016/j.jcis.2012.06.027.

(5) Tanaka, S.; Kaneti, Y. V.; Septiani, N. L. W.; Dou, S. X.; Bando, Y.; Hossain, Md. S. A.; Kim, J.; Yamauchi, Y. A Review on Iron Oxide-Based Nanoarchitectures for Biomedical, Energy Storage, and Environmental Applications. *Small Methods* **2019**, *3* (5), 1800512. https://doi.org/10.1002/smtd.201800512.

(6) Fruntke, A.; Behnke, M.; Stafast, L. M.; Träder, T.; Dietel, E.; Vollrath, A.; Weber, C.; Schubert, U. S.; Wilke, T. Targeted Drug Delivery: Synthesis of Smart Nanocarriers for School Chemistry Education. *J. Chem. Educ.* **2023**, *100* (2), 751–759. https://doi.org/10.1021/acs.jchemed.2c00422.

(7) Rattanakit, P. Open Inquiry-Based Laboratory Project on Plant-Mediated Green Synthesis of Metal Nanoparticles and Their Potential Applications. *J. Chem. Educ.* **2021**, *98* (12), 3984–3991. https://doi.org/10.1021/acs.jchemed.1c00300.

(8) Reddy, L. H.; Arias, J. L.; Nicolas, J.; Couvreur, P. Magnetic Nanoparticles: Design and Characterization, Toxicity and Biocompatibility, Pharmaceutical and Biomedical Applications. *Chem. Rev.* **2012**, *112* (11), 5818–5878. https://doi.org/10.1021/cr300068p.

(9) Lee, N.; Yoo, D.; Ling, D.; Cho, M. H.; Hyeon, T.; Cheon, J. Iron Oxide Based Nanoparticles for Multimodal Imaging and Magnetoresponsive Therapy. *Chem. Rev.* **2015**, *115* (19), 10637–10689. https://doi.org/10.1021/acs.chemrev.5b00112.

(10) Borys, A. M. An Illustrated Guide to Schlenk Line Techniques. *Organometallics* **2023**, *42* (3), 182–196. https://doi.org/10.1021/acs.organomet.2c00535.

(11) Scholtz, A.; Paulson, J.; Nunez, V.; Armani, A. M. Open-Source Magnetophotometer (MAP) for Nanoparticle Characterization. **2023**. https://doi.org/10.48550/arXiv.2401.01903.
