# Supplementary material for: Synthesis and Characterization of Superparamagnetic Iron Oxide Nanoparticles: A Series of Laboratory Experiments
Source: J Chem Educ. 2024 Apr 9;101(5):2039–44. doi: 10.1021/acs.jchemed.3c00996 (PMC11097384; doi:10.1021/acs.jchemed.3c00996)
Supplement: Supplementary file 6 — ed3c00996_si_006.docx [file ed3c00996_si_006.docx]

Supporting Information for:

**Synthesis and Characterization of Superparamagnetic Iron Oxide Nanoparticles: A Series of Laboratory Experiments**

Armando D. Urbina^1+^, Hari Sridhara^1+^, Alexis Scholtz^2+^, Andrea M. Armani^1,2,3^*

^1^ Mork Family Department of Chemical Engineering and Materials Science, University of Southern California, Los Angeles, CA 90089, USA

^2^ Alfred E. Mann Department of Biomedical Engineering, University of Southern California, Los Angeles, CA 90089, USA

^3^ Ellison Institute of Technology, Los Angeles, CA 90064, USA

^+^ These authors contributed equally.

[*aarmani@eit.org](mailto:*aarmani@eit.org)

**Synthesis and Characterization of Superparamagnetic**

**Iron Oxide Nanoparticles**

Student Assessment

Table of Contents

[Day 1 Pre-Lab 3](#_Toc158320693)

[Day 1 Overview 3](#_Toc158320694)

[Magnetism 4](#_Toc158320695)

[Synthetic Process 5](#_Toc158320696)

[Reagent Calculations 6](#_Toc158320697)

[Product Yield 6](#_Toc158320698)

[Reaction Fundamentals 7](#_Toc158320699)

[Day 2 Prelab 8](#_Toc158320700)

[Day 2 Overview 8](#_Toc158320701)

[Dynamic Light Scattering (DLS) 9](#_Toc158320702)

[Scanning Electron Microscopy (SEM) 10](#_Toc158320703)

[Ligand Exchange 11](#_Toc158320704)

[Day 3 Prelab 12](#_Toc158320705)

[Day 3 Overview 12](#_Toc158320706)

[DLS Analysis 13](#_Toc158320707)

[SEM Analysis 13](#_Toc158320708)

[Magnetophotometer (MAP) Analysis 14](#_Toc158320709)

Day 1 Pre-Lab

Day 1 Overview

On Day 1 of this lab, you will synthesize iron oxide nanoparticles and clean them via centrifugation (Figure S1). Prior to synthesizing these particles, you will need to determine the correct balanced chemical equation, calculate how much of each reagent you will need to run your reaction, calculate your expected product mass, and think about a few concepts related to the synthesis.


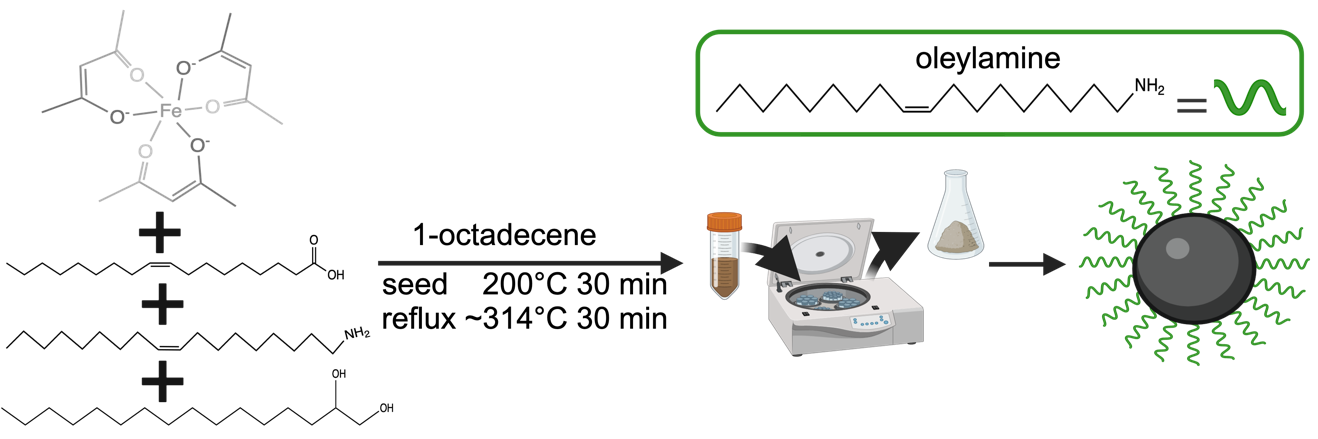


Figure S1. Overview of magnetic nanoparticle synthesis and cleaning.

Magnetism

1. One of the reasons magnetic nanoparticles are valued in research applications is because of their strong magnetic response. Explain the basis of the paramagnetic behavior in iron oxide particles.
2. How is this different from a ferromagnetic material such as a horseshoe magnet?

Synthetic Process

You will be synthesizing Fe_3_O_4_ nanoparticles. The following molar ratios should be used, assuming 20 mL of 1-octadecene:

- 2 mmol Fe(acac)_3_
- 5 mmol 1,2-hexadecanediol
- 6 mmol oleic acid
- 6 mmol oleylamine

1. For each reagent, write the molecular formula and draw the structure.
2. Write out the balanced chemical equation for this reaction. Be sure to label the reactants and products.

Reagent Calculations

1. Look up the molar mass of each reagent and the density of the liquid reagents. Then calculate the amount of each reagent required for a reaction volume of 80 mL of 1-octadecent. Solid reagents should be calculated by mass and liquid reagents should be calculated by volume. Fill in the following table. Be sure to include units for the calculated mass and volumes you will be using.

| **State** | **Reagent** | **Molar Mass [g/mol]** | **Num. Moles Needed** | **Density (Liquids Only) [g/mL]** | **Calculated Mass/Volume** |
| --- | --- | --- | --- | --- | --- |
| Solid | Fe(acac)_3_ |  |  | **---** |  |
| Solid | 1-2 hexadecanediol |  |  | **---** |  |
| Liquid | 1-octadecene |  |  |  |  |
| Liquid | Oleylamine |  |  |  |  |
| Liquid | Oleic acid |  |  |  |  |

Product Yield

1. Finally, calculate the expected mass of the product for your reaction. Assume this reaction has a 50% yield.

Reaction Fundamentals

1. What is the role of each of the reactants?
2. What is the purpose of each of the heating steps? What is the seed phase? What happens during reflux?

Day 2 Prelab

Day 2 Overview


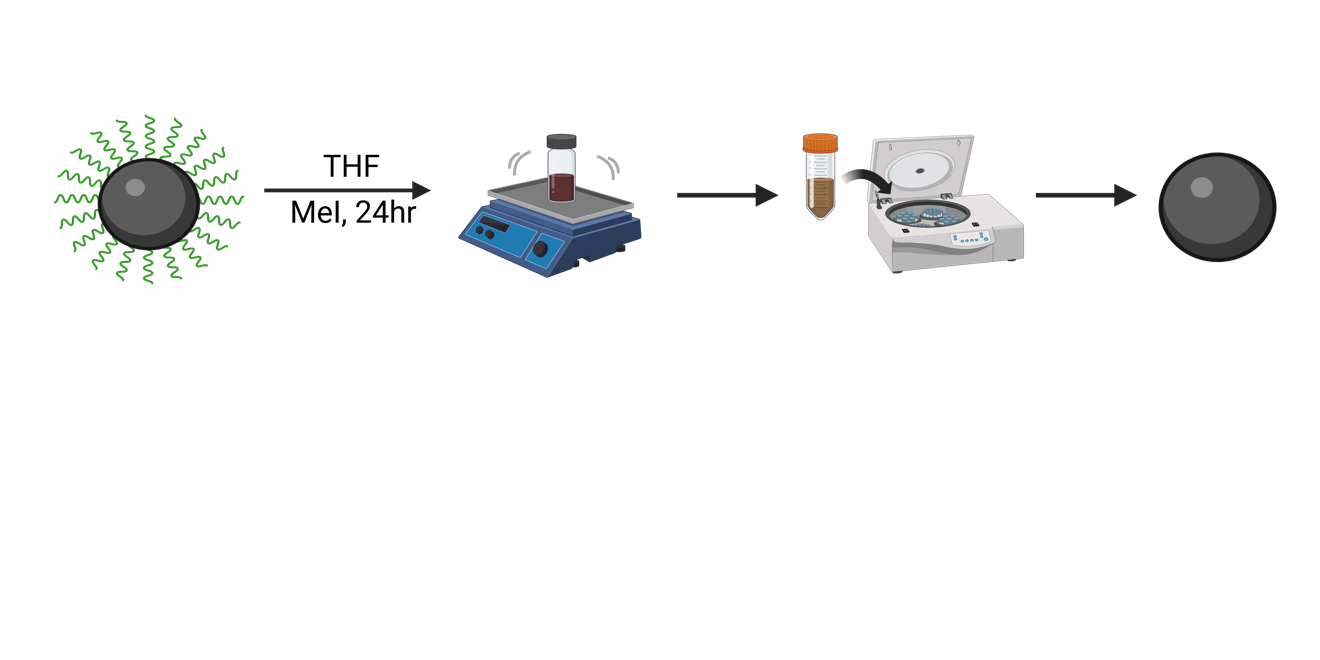
On Day 2 of this lab, you will strip the surface of the particles, perform a ligand exchange (Figure S2), and prepare samples for characterization on Day 3. Prior to this lab session, you will need to understand the principles behind the characterization methods and behind the ligand exchange.

Figure S2. Overview of the ligand exchange procedure.

Dynamic Light Scattering (DLS)

1. What information do we get from a DLS measurement?
2. Briefly describe the principle behind DLS. How are DLS measurements obtained?

Scanning Electron Microscopy (SEM)

1. How do scanning electron microscopes work?
2. What difficulties might we experience in imaging a magnetic material using an SEM? Propose a potential solution to this problem.
3. Why do some nanomaterials need to be sputter coated with a layer of gold before being imaged via SEM? Will your iron oxide nanoparticles require this?

Ligand Exchange

1. How do the oleylamine ligands on the surface of the nanoparticles change the polarity of the nanoparticle? Do they make the nanoparticles hydrophobic or hydrophilic? Explain your reasoning.
2. What role do the tetrahydrofuran (THF) and iodomethane play in the ligand exchange? How does the surface of the oleylamine-coated iron oxide nanoparticles become stripped?
3. Now that the nanoparticles are stripped, are they hydrophobic or hydrophilic? What would be a good solvent to dissolve them in? Keep in mind the solvent must also be compatible with PMMA cuvettes.

Day 3 Prelab

Day 3 Overview

On Day 3 of this lab, you will perform some of the characterization steps. A laboratory assistant will perform and provide you with DLS data prior to your lab session, and you will perform SEM measurements and magnetic characterization using a magnetophotometer (MAP) (Figure S3). Prior to this lab session, you will need to understand how to analyze DLS data, how to interpret an SEM image, and the principles behind the MAP.


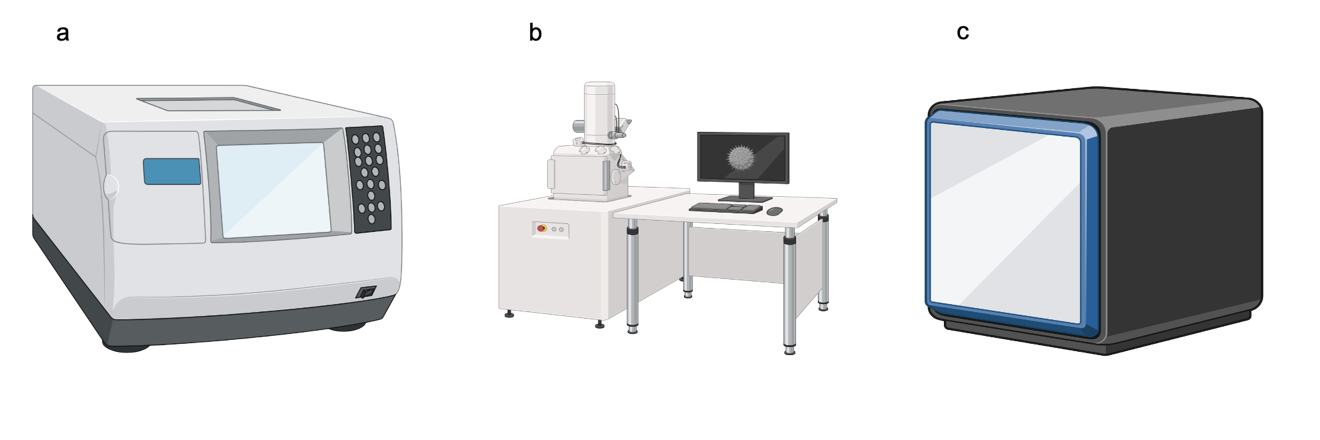


Figure S3. Characterization techniques used in this laboratory include (a) Dynamic Light Scattering (DLS), (b) Scanning Electron Microscopy (SEM), and (c) magnetophotometry (MAP).

DLS Analysis

1. What shape do we expect the measurement distribution to be for our single batch of nanoparticles? Explain your reasoning.
2. What shape would we expect the measurement distribution to be if we combined two batches of nanoparticles that had different reflux times (for example, one batch was heated in the reflux phase for 30 minutes and another batch was heated for 60 minutes)? Explain your reasoning.

SEM Analysis

1. If you image your iron oxide nanoparticles on a silicon wafer, which parts of the image do you expect to be lighter, and which would be darker? Explain your reasoning.

Magnetophotometer (MAP) Analysis

1. Consider just a single iron oxide nanoparticle in dispersion interacting with a magnet. What forces act upon that particle? Draw a force body diagram for a single particle. Assume that there are no forces in the xy (horizontal) directions and that forces only affect the particle in the z (vertical) direction.
2. Look up the definitions of paramagnetism and magnetic susceptibility and write them below (make sure to cite your sources). What does it mean when a material has a higher magnetic susceptibility than another material?
3. Similarly, to the question above, what is superparamagnetism?
4. When a paramagnetic (or superparamagnetic) particle is in dispersion and an external magnetic field is introduced, what happens to the particle? Your reasoning should consider the dipole of the particle in your analysis.
5. What will change between the responses of a dispersion of nanoparticles with a higher magnetic susceptibility and a dispersion of nanoparticles with lower magnetic susceptibility when a magnet is introduced underneath them? Think about how the particles will move in response to a magnetic field.
6. Now consider that we shine a light through the two dispersions and measure the power of the light that is transmitted through each. Sketch a plot of the light transmission over time for the two different dispersions on the same set of axes and identify the curves. The axes do not need to be quantitative, but they should show the general shape of the curve and demonstrate how the signal would change for the two different dispersions.
