# Supplementary material for: Synthesis and Characterization of Superparamagnetic Iron Oxide Nanoparticles: A Series of Laboratory Experiments
Source: J Chem Educ. 2024 Apr 9;101(5):2039–44. doi: 10.1021/acs.jchemed.3c00996 (PMC11097384; doi:10.1021/acs.jchemed.3c00996)

Supporting Information for:

Synthesis and Characterization of Superparamagnetic Iron Oxide Nanoparticles: A Series of Laboratory Experiments

Armando D. Urbina[1+], Hari Sridhara[1+], Alexis Scholtz[2+], Andrea M. Armani[1,2,3*]

[1] Mork Family Department of Chemical Engineering and Materials Science, University of Southern California, Los Angeles, CA 90089, USA

[2] Alfred E. Mann Department of Biomedical Engineering, University of Southern California, Los Angeles, CA 90089, USA

[3] Ellison Institute of Technology, Los Angeles, CA 90064, USA

[+] These authors contributed equally.

*aarmani@eit.org

Synthesis and Characterization of Superparamagnetic Iron Oxide Nanoparticles

Student Assessment Solutions

TABLE OF CONTENTS

Day 1 Pre-Lab *3*

Day 1 Overview 3

Magnetism 4

Synthetic Process 5

Reagent Calculations 7

Product Yield 7

Reaction Fundamentals 9

Day 2 Prelab *10*

Day 2 Overview 10

Dynamic Light Scattering (DLS) 11

Scanning Electron Microscopy (SEM) 12

Ligand Exchange 14

Day 3 Prelab *16*

Day 3 Overview 16

DLS Analysis 17

SEM Analysis 17

Magnetophotometer (MAP) Analysis 18

DAY 1 PRE-LAB

Day 1 Overview

On Day 1 of this lab, you will synthesize iron oxide nanoparticles and clean them via centrifugation (Figure S1). Prior to synthesizing these particles, you will need to determine the correct balanced chemical equation, calculate how much of each reagent you will need to run your reaction, calculate your expected product mass, and think about a few concepts related to the synthesis.

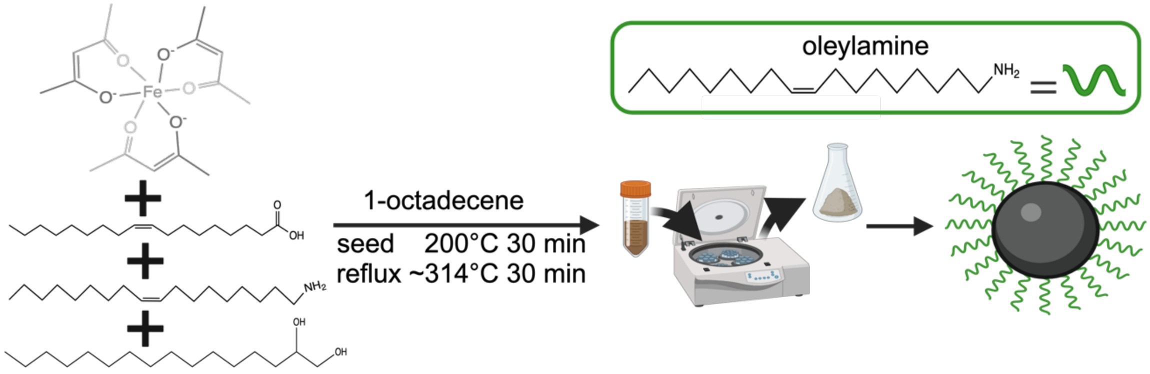

Figure S1. Overview of magnetic nanoparticle synthesis and cleaning.

Magnetism

1. One of the reasons magnetic nanoparticles are valued in research applications is because of their strong magnetic response. Explain the basis of the paramagnetic behavior in iron oxide particles.

   Materials contain individual magnetic dipoles and can be classified into different magnetic material classes based on the orientation of these dipoles in the presence and absence of external magnetic fields. In paramagnetic materials, these dipoles are naturally randomly oriented in the absence of an external magnetic field, but if an external magnetic field is present, the dipoles will align themselves within the material and be attracted to the external magnetic field.

2. How is this different from a ferromagnetic material such as a horseshoe magnet?

   In ferromagnets, these magnetic dipoles form larger magnetic domains. Within each domain, the magnetic dipoles are naturally aligned to produce what is commonly referred to as a "permanent" magnetic field. Note that these domains can be disrupted under select conditions such as extremely high temperatures, which disrupts the magnetic field of the ferromagnet.

Synthetic Process

You will be synthesizing Fe_3O_4 nanoparticles. The following molar ratios should be used, assuming 20 mL of 1-octadecene:

- 2 mmol $Fe(acac)_3$
- 5 mmol 1,2-hexadecanediol
- 6 mmol oleic acid
- 6 mmol oleylamine

3. For each reagent, write the molecular formula and draw the structure.

Fe(III)(acac)$_3$: $Fe(C_5H_7O_2)_3$

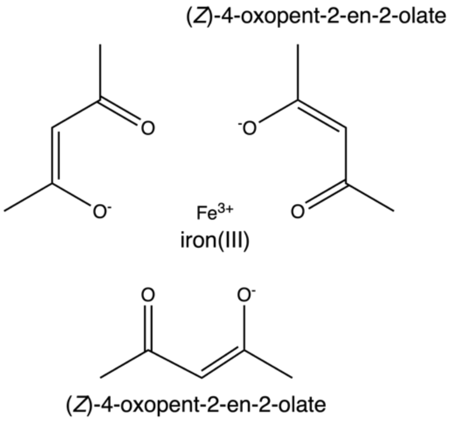

Tris(acetylacetonato)iron(III)

1,2-hexadecanediol: $CH_3(CH_2)_{13}CHOHCH_2OH$

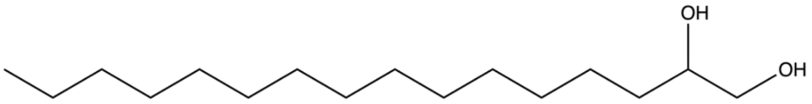

1,2-hexadecanediol

Oleic acid: $C_{18}H_{34}O_2$

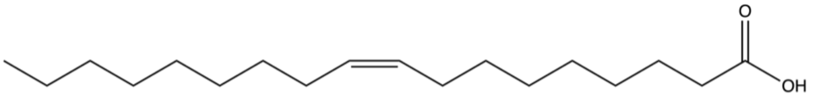

oleic acid

Oleylamine: $C_{18}H_{37}NH_2$

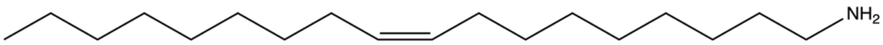

oleylamine

4. Write out the balanced chemical equation for this reaction. Be sure to label the reactants and products.

$$3Fe(C_5H_7O_2)_3 + 2C_{16}H_{34}O_2 \xrightleftharpoons[\text{oleic acid, oleylamine}]{\text{1-octadecene}} Fe_3O_4$$

Reagents are: $Fe(III)(acac)_3$, 1,2-hexadecanediol, oleic acid, and oleylamine.

Our reaction solvent is 1-octadecene.

The product is Fe_3O_4:oleylamine.

Reagent Calculations

5. Look up the molar mass of each reagent and the density of the liquid reagents. Then calculate the amount of each reagent required for a reaction volume of 80 mL of 1-octadecene. Solid reagents should be calculated by mass and liquid reagents should be calculated by volume. Fill in the following table. Be sure to include units for the calculated mass and volumes you will be using.

State	Reagent	Molar Mass [g/mol]	Num. Moles Needed [mmol]	Density (Liquids Only) [g/mL]	Calculated Mass/Volume
Solid	$Fe(acac)_3$	353.17	8	---	2.83 g
Solid	1-2 hexadecanediol	258.44	20	---	5.17 g
Liquid	1-octadecene	252.48	250	0.789	80 mL
Liquid	Oleylamine	267.49	24	0.813	7.90 mL
Liquid	Oleic acid	282.47	24	0.895	7.57 mL

Product Yield

6. Finally, calculate the expected mass of the product for your reaction. Assume this reaction has a 50% yield.

To calculate ideal 100% yield: grams $Fe(acac)_3$ → moles $Fe(acac)_3$ → moles Fe_3O_4 → grams Fe_3O_4. The students will need to reference the balanced chemical equation from question 4:

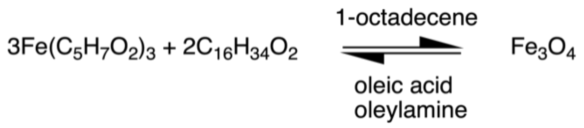

If this reaction has 100% yield, 2.85 grams of $Fe(acac)_3$ would yield 0.6175 grams of Fe_3O_4. So if this reaction has 50% yield, 2.85 grams of $Fe(acac)_3$ would yield 0.309 grams Fe_3O_4.

Reaction Fundamentals

7. What is the role of each of the reactants?

Fe(III)acetylacetonate is iron source for iron oxide nanoparticles.

The 1,2-hexadecanediol serves to reduce the Fe(III)acetylacetonate into constituent iron and acetylacetonate ions.

The oleic acid precursor serves two purposes: (1) to provide oxygen groups used in forming Fe_3O_4 particles and (2) to protect the nanoparticles from colliding into each other during growth.

The nitrogen group on oleylamine coordinates with the iron oxide surface to form a surface-protecting group. This protecting group keeps particles form clumping with each other and mitigates oxidation.

8. What is the purpose of each of the heating steps? What is the seed phase? What happens during reflux?

The purpose of the seed phase is to provide sufficient heat to for 1,2-hexadeacanediol to cleave Fe^{3+} from acetylacetonate.

The additional heat added into the system during the reflux phase makes growth of the nucleated iron oxide particles preferential over formation of new particles.

It is important to use a combination of seed and reflux steps to maintain control over the size of the particles produced during the reaction.

DAY 2 PRELAB

Day 2 Overview

On Day 2 of this lab, you will strip the surface of the particles, perform a ligand exchange (Figure S2), and prepare samples for characterization on Day 3. Prior to this lab session, you will need to understand the principles behind the characterization methods and behind the ligand exchange.

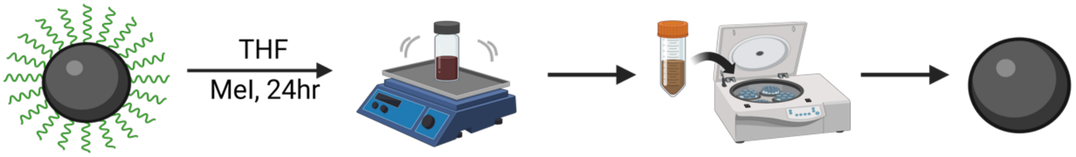

Figure S2. Overview of the ligand exchange procedure.

Dynamic Light Scattering (DLS)

1. What information do we get from a DLS measurement?

   DLS measurements provide quantitative information regarding the size distribution of iron oxide particles in a dispersion. Specifically, using the DLS data, the hydrodynamic radius and size distribution the particles can be calculated.

2. Briefly describe the principle behind DLS. How are DLS measurements obtained?

   During DLS measurements, the scattering of light is measured as it passes through the particle dispersion. Brownian motion of the dispersed particles leads to fluctuations in the scattered light, and the rate and intensity of these fluctuations are related to the size of the particles. The light signals are processed by an internal correlation function that outputs information about the particle size.

   Additional information is available at: *Dynamic Light Scattering: An Introduction in 30 Minutes*, warwick.ac.uk/fac/cross_fac/sciencecity/programmes/internal/themes/am2/booking/particlesize/intro_to_dls.pdf. Accessed 5 Dec. 2023.

SCANNING ELECTRON MICROSCOPY (SEM)

3. How do scanning electron microscopes work?

A sample is put inside a vacuum chamber and electrons are fired from a source, manipulated into a beam using a series of electromagnets, and aimed at a target specimen. The electron beam scans the sample in a serial fashion and the charged detector gathers the reflected electrons.

Additional information is available at: Ford, Brian J. , Joy, David C. and Bradbury, Savile. "scanning electron microscope". Encyclopedia Britannica, 12 Oct. 2023, https://www.britannica.com/technology/scanning-electron-microscope.

4. What difficulties might we experience in imaging a magnetic material using an SEM? Propose a potential solution to this problem.

One possible answer is that the electron beam in a SEM will induce a magnetic field that may attract iron oxide nanoparticles. To mitigate particle powder attraction, it is necessary to secure the particles utilizing a conductive adhesive tape such as carbon or copper tape.

Another possible difficulty when imaging these particles would be that oleylamine, the surface protecting ligand, is a long-chain hydrocarbon and is prone to charging. When imaging insulating materials such as oleylamine, one might experience charging, which causes a buildup of current and will lead to persistent white areas in the material. Thus, it is important to ground the samples when imaging.

5. Why do some nanomaterials need to be sputter coated with a layer of gold before being imaged via SEM? Will your iron oxide nanoparticles require this?

   It is necessary to sputter coat a thin conducting layer onto samples made of insulating materials. While our stripped iron oxide particles have a higher resistance than iron metal, they are sufficiently conducting to not need sputter coating.

   If we were to try to image iron oxide particles coated with oleylamine, sputter coating with a conductive material such as gold would be necessary.

Ligand Exchange

6. How do the oleylamine ligands on the surface of the nanoparticles change the polarity of the nanoparticle? Do they make the nanoparticles hydrophobic or hydrophilic? Explain your reasoning.

   The uncoated iron oxide particles will have many OH^- groups on the surface which makes the particles more hydrophilic.
   On the other hand, oleylamine is an unsaturated fatty amine which is fairly stable on the surface of iron oxide particles. The hydrogen atoms in this compound are tightly bound which leads to a non-polar compound with highly hydrophobic behavior. One reason that students might give for this hydrophobic behavior is that there are no oxygens in this material. While that rationale is partially correct there could be other functional groups that contribute to hydrophilic behavior. Another possible explanation for the hydrophobic behavior is the long carbon chain attached to the amine group. The structure of this molecule resembles oils which students might (correctly) associate with non-polar compounds.

7. What role do the tetrahydrofuran (THF) and iodomethane play in the ligand exchange? How does the surface of the oleylamine-coated iron oxide nanoparticles become stripped?

   The iodine in iodomethane is a good leaving group, which allows us to use this molecule to methylate the amine group on oleylamine. The result of iodomethane treatment is to efficiently strip oleylamine from the surface of our iron oxide particles.
   THF is a polar organic solvent which is compatible with both the oleylamine-coated particles, iodomethane, and the stripped iron oxide particles, allowing us to keep the particles dissolved in both their stripped and unstripped states.

8. Now that the nanoparticles are stripped, are they hydrophobic or hydrophilic? What would be a good solvent to dissolve them in? Keep in mind the solvent must also be compatible with PMMA cuvettes.

   The uncoated iron oxide particles will have many OH^- groups on the surface which makes the particles more hydrophilic.

   Depending on the solvents available in the classroom the solutions will vary. Some common solvents that could be possible answers are hexane, isopropanol, heptane.

   This question can be modified depending on available cuvettes.

DAY 3 PRELAB

Day 3 Overview

On Day 3 of this lab, you will perform some of the characterization steps. A laboratory assistant will perform and provide you with DLS data prior to your lab session, and you will perform SEM measurements and magnetic characterization using a magnetophotometer (MAP) (Figure S3). Prior to this lab session, you will need to understand how to analyze DLS data, how to interpret an SEM image, and the principles behind the MAP.

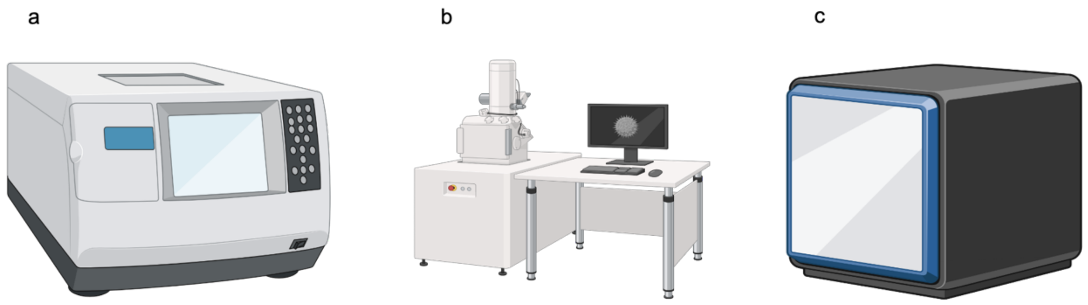

Figure S3. Characterization techniques used in this laboratory include (a) Dynamic Light Scattering (DLS), (b) Scanning Electron Microscopy (SEM), and (c) magnetophotometry (MAP).

DLS Analysis

1. What shape do we expect the measurement distribution to be for our single batch of nanoparticles? Explain your reasoning.

   We expect to have relatively uniform nanoparticles within a single size population. The distribution is expected to be a narrow Gaussian distribution.

2. What shape would we expect the measurement distribution to be if we combined two batches of nanoparticles that had different reflux times (for example, one batch was heated in the reflux phase for 30 minutes and another batch was heated for 60 minutes)? Explain your reasoning.

   With two distinct populations of nanoparticles, we would expect to see a bimodal distribution of sizes from the DLS measurements. A longer reflux time will increase the size of the particles because they are allowed to react longer, so the population at the larger size corresponds to that respective batch. Note that the relative sizes of the two modes within the distribution would correspond to the relative ratios of the number of nanoparticles in each batch.

SEM Analysis

3. If you image your iron oxide nanoparticles on a silicon wafer, which parts of the image do you expect to be lighter, and which would be darker? Explain your reasoning.

   When we are imaging uncoated iron oxide particles with an appropriate grounding wire, we should not experience significant charging. In this case, our iron oxide will appear brighter than the silicon wafer because the element is heavier.

Magnetophotometer (MAP) Analysis

4. Consider just a single iron oxide nanoparticle suspended in solution interacting with a magnet. What forces act upon that particle? Draw a force body diagram for a single particle. Assume that there are no forces in the xy (horizontal) directions and that forces only affect the particle in the z (vertical) direction.

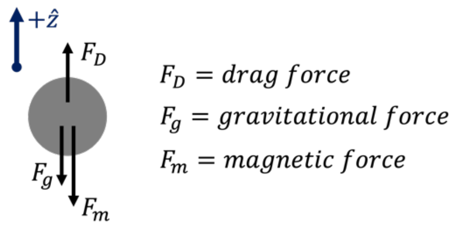

5. Look up the definitions of paramagnetism and magnetic susceptibility and write them below. What does it mean when a material has a higher magnetic susceptibility than another material?

   Paramagnetism is a material property in which the magnetic dipoles within a material align to an external magnetic field such that the material is attracted to the magnet only when an external field is present and are otherwise randomly oriented.
   Magnetic susceptibility is a measure of how magnetized a material becomes in response to an external magnetic field.

6. What is superparamagnetism?

   Superparamagnetic materials exhibit superior magnetic properties, including short magnetic relaxation times and no remnant magnetization when an external magnetic field is removed. This property can be exhibited by nanoparticles small enough that they contain only a single magnetic dipole.

7. When a paramagnetic (or superparamagnetic) particle is suspended in solution and an external magnetic field is introduced, what happens to the particle? Your reasoning should consider the dipole of the particle in your analysis.

The dipoles (or single dipole if superparamagnetic) of the particle will align themselves to the external magnetic field, rotating the particle. Because the particle is suspended in solution and can freely move, it will move through solution towards the magnet until it is stopped by the wall of the vessel.

8. What will change between the responses of a dispersion of nanoparticles with a higher magnetic susceptibility and a dispersion of nanoparticles with lower magnetic susceptibility when a magnet is introduced underneath them? Think about how the particles will move in response to a magnetic field.

Nanoparticles with a higher magnetic susceptibility will respond more strongly to the external magnetic field. Therefore, in dispersions of these nanoparticles, the nanoparticles will be aggregated in solution close to the source of the magnetic field more quickly than nanoparticles with lower magnetic susceptibility would.

9. Now consider that we shine a light through the two dispersions and measure the power of the light that is transmitted through the dispersions. Sketch a plot of the light transmission over time for the two different dispersions on the same set of axes and identify the curves. The axes do not need to be quantitative, but they should show the general shape of the curve and demonstrate how the signal would change for the two different dispersions.

The light transmission should follow a logarithmic shape that eventually plateaus that represents changes in transmission due to the nanoparticles being pulled to the bottom of the solution vessel. The light transmission signal would plateau at the same point, assuming the two concentrations are the same. However, the slope of the curves should change; a dispersion of nanoparticles with a high magnetic susceptibility (χ) (blue) will have a sharper increase in light transmission than a dispersion of nanoparticles with a lower χ (green). Note that on the plot below, a conceptual curve for non-magnetic particles (orange) is also included; this would not have the characteristic curve shape of the magnetic particles.

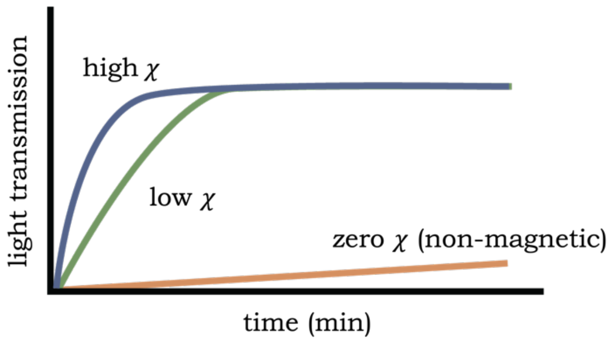

Supplement: Supplementary file 7 — ed3c00996_si_007.pdf [file ed3c00996_si_007.pdf]
